# Natural Compounds and Their Structural Analogs in Regio- and Stereoselective Synthesis of New Families of Water-Soluble 2*H*,3*H*-[1,3]thia- and -Selenazolo[3,2-*a*]pyridin-4-ium Heterocycles by Annulation Reactions

**DOI:** 10.3390/molecules25020376

**Published:** 2020-01-16

**Authors:** Vladimir A. Potapov, Roman S. Ishigeev, Irina V. Shkurchenko, Sergey V. Zinchenko, Svetlana V. Amosova

**Affiliations:** 1 A. E. Favorsky Irkutsk Institute of Chemistry, Siberian Division of The Russian Academy of Sciences, 1 Favorsky Str., Irkutsk 664033, Russian; ishigeev@irioch.irk.ru (R.S.I.); mindofchem@gmail.com (I.V.S.); svz@irioch.irk.ru (S.V.Z.); amosova@irioch.irk.ru (S.V.A.); 2 Pedagogical Institute, Irkutsk State University, 6 Nizhniaja Naberezhnaya Str., Irkutsk 664003, Russia

**Keywords:** anethole, eugenol, isoeugenol, 2-pyridinesulfenyl halides, 2-pyridineselenenyl halides, [1,3]thiazolo[3,2-*a*]pyridin-4-ium derivatives, [1,3]selenazolo[3,2-*a*]pyridin-4-ium derivatives

## Abstract

It has been found that both eugenol and isoeugenol derivatives reacted with 2-pyridinesulfenyl and 2-pyridineselenenyl halides in a regioselective mode affording products with opposite regiochemistry. Synthesis of new families of 2*H*,3*H*-[1,3]thia- and -selenazolo[3,2-*a*]pyridin-4-ium heterocycles has been developed by annulation reactions of 2-pyridinechalcogenyl halides with natural compounds (eugenol, isoeugenol, methyl eugenol, methyl isoeugenol, acetyl eugenol, trans-anethole) and their structural analogs. The influence of the substrate structure and the nature of halogen on the product yields are studied. The 2-pyridinesulfenyl and 2-pyridineselenenyl chlorides are more efficient reagents compared to corresponding bromides. The obtained condensed heterocycles are novel water-soluble functionalized compounds with promising biological activity.

## 1. Introduction 

**Figure 1:**
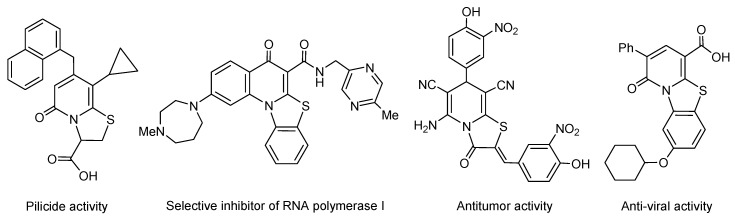
Known biologically active compounds structurally related to the [1,3]thiazolo[3,2-a]pyridinium scaffold.

Sulfur-containing rings condensed with nitrogen heterocycles are important scaffolds for medicinal chemistry [1]. Condensed sulfur/nitrogen heterocycles have always been a vital part of new drug discovery. For example, penicillin and cephalosporin antibiotics contain sulfur-containing ring condensed with a nitrogen heterocycle as the key scaffold [1]. Thiazole ring presents in many natural and biologically active compounds including ritonavir (anti-HIV), sulfathiazole (antimicrobial), and tiazofurin (anticancer) drugs [1,2,3]. The combination of the thiazole ring with the pyridine heterocycle in one condensed molecule gives a very promising scaffold for medicinal chemistry. The thiazolopyridine derivatives are associated with a wide range of pharmacological activities. Among them, [1,3]thiazolo[3,2-*a*]pyridinium derivatives and structurally related compounds exhibit antitumor, antiviral, antibacterial, pilicide activity and properties of selective inhibitor of RNA polymerase I [4,5,6,7,8] (Figure 1). Selenium analogs, [1,3]selenazolo[3,2-*a*]pyridinium derivatives are less studied. Selenium-containing heterocycles [9,10,11,12,13] represent an important family of compounds exhibiting various types of biological activity including anti-inflammatory, antitumor, antifungal, and glutathione peroxidase-like action [12,13,14,15,16,17,18,19,20]. The selenium-containing heterocyclic drug ebselen is applied for the prevention and treatment of human ischemic stroke [21]. The development of effective approaches to novel selenium heterocycles by regioselective cyclization and annulation reactions is one of the main directions of our research [22,23,24,25,26,27,28,29,30,31].

The reactions of 2-pyridinesulfenyl and 2-pyridineselenenyl chlorides with some alkenes have been described [32,33,34,35,36,37,38,39,40,41]. The reactions of these reagents with styrene have been reported to afford 3-phenyl-2*H*,3*H*-[1,3]thiazolo- and -selenazolo[3,2-а]pyridin-4-ium chlorides in high yields [32,35]. In spite of some progress in synthetic method for preparations of [1,3]thiazolo[3,2-*a*]- and [1,3]selenazolo[3,2-*a*]pyridin-4-ium derivatives [32,33,34,35,36,37,38,39,40,41], reactions of 2-pyridinesulfenyl and 2-pyridineselenenyl halides with many alkenes are hitherto unknown. For example, the reactions of 2-pyridinesulfenyl and 2-pyridineselenenyl chlorides with simple 1-alkenes and natural compounds containing a double bond such as eugenol, isoeugenol, anethole and its derivatives have not been described. Besides, the influence of the substrate structure and the nature of halogen on the product yields in the reaction of 2-pyridinechalcogenyl halides with alkenes is hitherto unknown.

The developments of synthetic approaches to novel derivatives of [1,3]thiazolo[3,2-*a*]- and [1,3]selenazolo[3,2-*a*]pyridin-4-ium scaffolds based on functionalized alkenes including natural products and studies of their properties remains an important task. 

Chemistry of natural products is very important for providing knowledge about medicines to derive active components as lead compounds for drug discovery. It is known that the majority of new drugs have been developed from natural products and synthesis of novel compounds based on natural products is prospective for searching biologically active substances. 

The goal of the present research is the development of regio- and stereoselective synthesis of novel condensed heterocycles with promising biological activity based on the annulation reactions of 2-pyridinesulfenyl and 2-pyridineselenenyl halides with natural compounds (eugenol, isoeugenol, anethole) and their derivatives and structural analogs as well as studies on the influence of the substrate structure and the nature of halogen and chalcogen on the product yields.

## 2. Results and Discussion

**Scheme 1:**
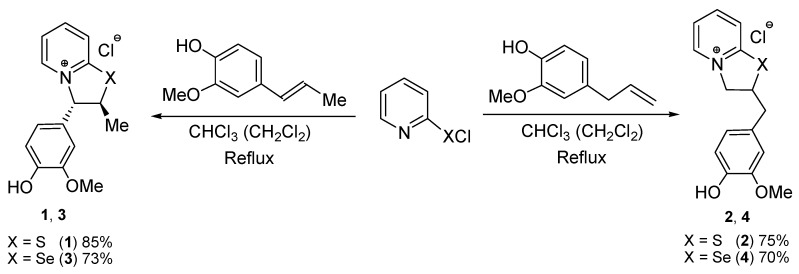
Synthesis of compounds **1**–**4** based on the reactions of 2-pyridinesulfenyl and 2-pyridineselenenyl chlorides eugenol and isoeugenol.

Recently we attempted two representative of phenylpropanoids, eugenol (4-allyl-2-methoxyphenol) and isoeugenol (2-methoxy-4-propenylphenol), as substrates in the annulation reactions with 2-pyridinesulfenyl and -selenenyl chlorides. The reactions of 2-pyridinesulfenyl chloride with isoeugenol leading to 3-(3,4-dimethoxyphenyl)-2-methyl-2*H*,3*H*-thiazolo[3,2-*а*]pyridin-4-ium chloride (**1**) in 70% yield was reported by us as a short letter [41]. In the present research, we increased the yield of compound **1** to 85% and proved that compound **1** has trans-configuration regarding the positions of methyl substituent and benzene ring with respect to the condensed thiazolo[3,2-*а*]pyridin-4-ium bicycle (Scheme 1). 

The reaction of 2-pyridinesulfenyl chloride with eugenol (an equimolar ratio of the reagents) after overnight stirring (20 h) at room temperature in methylene chloride led to 2-[(4-hydroxy-3-methoxyphenyl)methyl]-*2H,3H*-[1,3]thiazolo[3,2-*а*]pyridin-4-ium chloride (**2**) (34% yield), which was precipitated from the reaction mixture as a powder. Similar result was obtained using chloroform as a solvent instead of methylene chloride. The yield was increased to 75% by refluxing the mixture of 2-pyridinesulfenyl chloride with eugenol in chloroform (Scheme 1). 

Compounds **1** and **2** can be regarded as the products with the opposite regiochemistry with respect to the location of aryl-containing substituents: compound **1** has aryl on the position 3 of the dihydrothiazole ring whereas aryl-containing substituent is situated on the position 2 in compound **2**. Thus, the reactions of 2-pyridinesulfenyl chloride with both eugenol and isoeugenol proceeded under similar conditions in a regioselective mode giving products **1** and **2** with the opposite regiochemistry (Scheme 1). 

This interesting result inspired us to study carefully the reactions of 2-pyridinesulfenyl and 2-pyridineselenenyl chlorides with eugenol and isoeugenol derivatives. Some other natural products (trans-anethole, (1*S*)-(−)-beta-pinene) were also investigated in reactions with 2-pyridinechalcogenyl chlorides.

The reactions of 2-pyridineselenenyl chloride with isoeugenol and eugenol under the same conditions as the synthesis of compound **1** afforded trans-3-(3,4-dimethoxyphenyl)-2-methyl-2*H*,3*H*-[1,3]selenazolo[3,2-*а*]pyridin-4-ium chloride (**3**) and 2-[(4-hydroxy-3-methoxyphenyl)methyl]-*2H*,3*H*-[1,3]selenazolo[3,2-*а*]pyridin-4-ium chloride (**4**) in 73% and 70% yields, respectively (Scheme 1). 

Like the synthesis of compounds **1** and **2,** the reactions of 2-pyridineselenenyl chloride with eugenol and isoeugenol led to two structural isomers of the opposite regiochemistry, compounds **3** and **4**, selenium analogs of heterocycles **1** and **2**. 

**Scheme 2:**
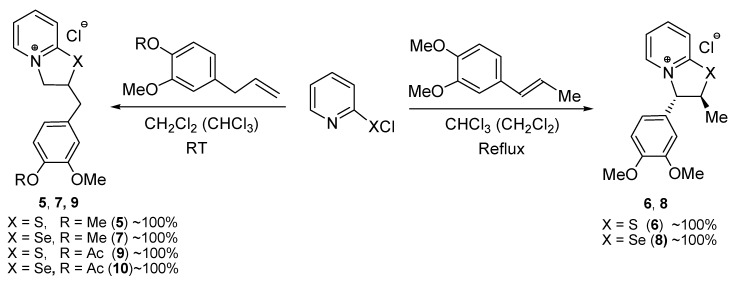
Synthesis of compounds **5**–**10** from eugenol and isoeugenol derivatives.

The reactions of 2-pyridinesulfenyl chloride with methyl eugenol and methyl isoeugenol were found to be more efficient compared to those with eugenol and isoeugenol. The reaction of 2-pyridinesulfenyl chloride with methyl eugenol (an equimolar ratio of the reagents) proceeded smoothly at room temperature in methylene chloride giving 2-[(3,4-dimethoxyphenyl)methyl]-*2H,3H*-[1,3]thiazolo[3,2-*а*]pyridin-4-ium chloride (**5**) in quantitative yield (Scheme 2). 

The reaction of 2-pyridinesulfenyl chloride with methyl isoeugenol occurred at reflux temperature in chloroform affording trans-3-(3,4-dimethoxyphenyl)-2-methyl-*2H*,*3H*-[1,3]thiazolo[3,2-*а*]pyridin-4-ium chloride (**6**) in quantitative yield (Scheme 2). Unlike the reactions with eugenol and isoeugenol (Scheme 1), the precipitation of the products **5** and **6** from the reaction mixture was not observed. The compounds **5** and **6** are two structural isomers with the opposite regiochemistry.

Similar results were obtained using 2-pyridineselenenyl chloride. This reagent reacted with methyl eugenol and methyl isoeugenol very smoothly under the same conditions as the synthesis of heterocycles **5** and **6** leading to the selenium analogs of these compounds in quantitative yields (Scheme 2). The obtained condensed selenium heterocycles **7** and **8** are also two structural isomers with the opposite regiochemistry. 

Acetyleugenol was involved in the reactions with 2-pyridinesulfenyl and 2-pyridineselenenyl chlorides. The reactions occurred at room temperature in methylene chloride giving 2-[[4-(acetyloxy)-3-methoxyphenyl]methyl]-*2H,3H*-[1,3]thiazolo- and -selenazolo[3,2-*а*]pyridin-4-ium chlorides **9** and **10** (acetyl analogs of products **2** and **4**) in quantitative yields (Scheme 2). 

A complex mixture of products was obtained in the reactions of 2-pyridinesulfenyl and 2-pyridineselenenyl chlorides with (1*S*)-(−)-beta-pinene. 

**Scheme 3:**
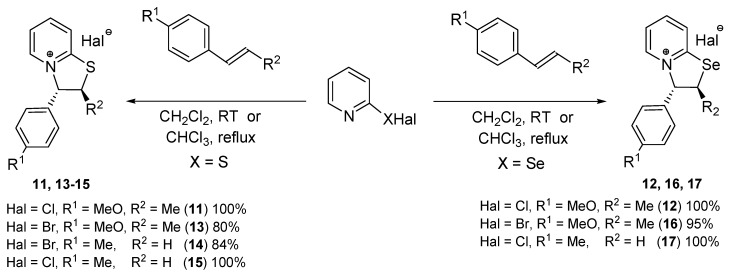
Synthesis of compounds **11**–**17** from structural analogs of isoeugenol.

Structural analogs of eugenol and isoeugenol were involved in the reactions of 2-pyridinesulfenyl and 2-pyridineselenenyl halides. trans-Anethole, trans-1-methoxy-4-(1-propenyl)benzene, is the structural analog of isoeugenol. This compound was found to react with 2-pyridinesulfenyl and 2-pyridineselenenyl chlorides giving trans-3-(4-methoxyphenyl)-2-methyl-2*H*,3*H*-[1,3]thiazolo- and selenazolo[3,2-*а*]pyridin-4-ium chlorides **11** and **12** in quantitative yields (Scheme 3).

2-Pyridinesulfenyl bromide and 2-pyridineselenenyl bromide were rarely used in organic synthesis and in the preparation of condensed heterocycles [37,38,39]. Usually organylsulfenyl and organylselenenyl bromides are less electrophilic than corresponding organylsulfenyl and organylselenenyl chlorides. On the other hand, the bromine atom is more reactive in the nucleophilic substitution reactions compared to the chlorine atom and can be easily substituted with nitrogen atom of the pyridine ring forming condensed heterocycles. 

We studied the reactions of 2-pyridinesulfenyl and 2-pyridineselenenyl bromides with a series of substrates and found that, in general, these reagents are less efficient in the annulation reactions compared to corresponding chlorides and the yields of target products are higher in the case of 2-pyridinesulfenyl or 2-pyridineselenenyl chlorides. 

The reactions of 2-pyridinesulfenyl bromide with anethole and 4-methylstyrene afforded compounds **13** and **14** in 80 and 84% yields, whereas quantitative yields of chloro analogs **11** and **15** were achieved with these substrates using 2-pyridinesulfenyl chloride (Scheme 3).

The selenium analog of heterocycle **15** was obtained in quantitative yield from 2-pyridineselenenenyl chloride and 4-methylstyrene (compound **17**, Scheme 3).

**Scheme 4:**
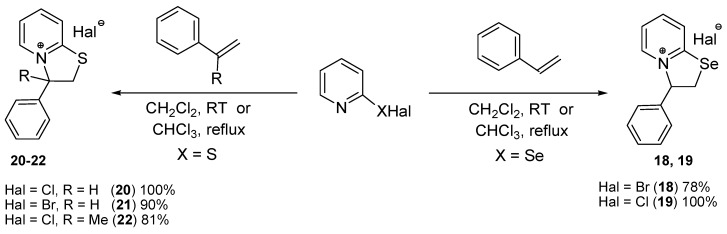
Synthesis of compounds **18**–**22** from styrene and its derivative.

The reactions of 2-pyridineselenenyl bromide with trans-anethole and styrene led to heterocycles **16** and **18** in 95% and 78% yields, respectively; however, analogous reactions of 2-pyridineselenenyl chloride with these substrates afforded target products **12** and **19** in quantitative yields (Scheme 3 and Scheme 4). In the case of 2-pyridinesulfenyl bromide, the reaction with styrene gave product **21** in 90% yield, whereas the formation of heterocycle **20** quantitative yield was observed in the reaction of 2-pyridinesulfenyl chloride with styrene (Scheme 4).

These results indicate that, in general, 2-pyridinesulfenyl and 2-pyridineselenenyl chlorides are more efficient compared to corresponding bromides and the annulation reactions of 2-pyridinechalcogenyl chlorides afforded the desired products in higher (mostly quantitative) yields. 

The introduction of methyl substituent at β-position of the double bond of styrene as well as to the position 4 of the benzene ring has little influence on the yields of products in annulation reactions. However, the introduction of methyl substituent at α-position of the double bond of styrene seems to have negative effect on the annulation process. The reaction of 2-pyridinesulfenyl chloride with α-methylstyrene led to heterocycle **22** only in 81% yield (Scheme 4). A mixture of products was obtained in the reaction of 2-pyridineselenenyl chloride with α-methylstyrene, whereas product **19** was formed in quantitative yield in the reaction of 2-pyridineselenenyl chloride with styrene (Scheme 4). Some decrease in efficiency of the annulation reaction in the case of α-methylstyrene can be attributed to steric factor: the introduction of the methyl group diminishes the rate of nucleophilic substitution by the nitrogen atom of the pyridine ring in the last stage.

**Scheme 5:**
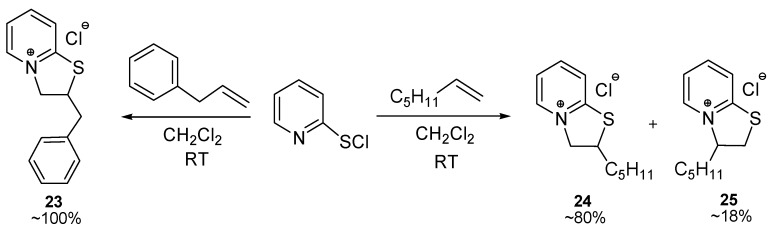
The reactions of 2-pyridinesulfenyl chloride with allylbenzene and 1-heptene.

Allylbenzene reacted with 2-pyridinesulfenyl chloride at room temperature in a regioselective mode affording heterocycle **23** (quantitative yield) derived from anti-Markovnikov addition to the double bond (Scheme 5). Thus, allylbenzene derivatives (eugenol, methyl eugenol, acetyleugenol) and styrene derivatives (isoeugenol, methyl isoeugenol, trans-anethole) reacted with 2-pyridinesulfenyl and 2-pyridineselenenyl halides in a regioselective mode affording products with the opposite regiochemistry with respect to the location of aryl-containing substituents. 

**Scheme 6:**
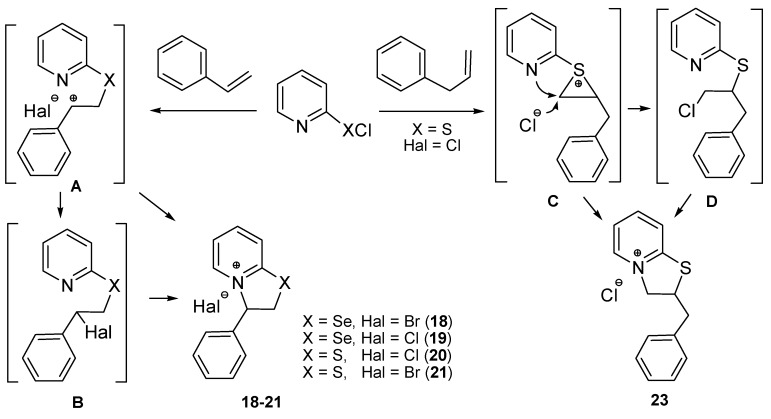
The suggested reactions pathways on the example of reactions of 2-pyridinesulfenyl and selenenyl halides with styrene and allylbenzene.

Suggested reactions pathways can be regarded on the examples of reactions of 2-pyridinechalcogenyl halides with styrene and allylbenzene (Scheme 6). The reactions of 2-pyridinesulfenyl and selenenyl halides with compounds containing a double bond conjugated with the benzene ring (isoeugenol, methyl isoeugenol, trans-anethole, styrene, 4-methylstyrene, α-methylstyrene) proceed regioselectively via electrophilic addition of the chalcogen atom at β-carbon atom of the double bond. The regioselectivity is due to the formation of intermediate carbocation **A**, which is stabilized by the benzene ring (the relatively stable benzyl cation) (Scheme 6, **B** is also possible intermediate). Noteworthy, the known addition reactions of arylsulfenyl and arylselenenyl halides to styrene and its derivatives also afforded Markovnikov adducts [42,43].

In the case of eugenol, its derivatives and structural analogs, 2-pyridinesulfenyl and selenenyl halides react with allyl group as with linear 1-alkene and electrophilic addition of the chalcogen atom occurs at α-carbon atom of the double bond (**C** and **D** are possible intermediates in the reaction of 2-pyridinesulfenyl chloride with allylbenzene) followed by intramolecular nucleophilic substitution in the formed anti-Markovnikov adduct (Scheme 6). It is known that electrophilic addition of sulfenyl halides to linear 1-alkenes afforded predominantly anti-Markovnikov products [44,45,46,47]. Thiiranium [45,46,47,48] and seleniranium [47,48,49,50,51,52,53,54] cations are often regarded as intermediates in electrophilic addition of chalcogenyl halides to the double bond, and attack of the halide anion occurs at unsubstituted carbon atom of thiiranium or seleniranium cations leading to anti-Markovnikov products. Besides, the formation of thiiranium and seleniranium species determines the reaction course as anti-addition in reactions of sulfenyl and selenenyl halides with alkenes. For example, the known reactions of arylsulfenyl and arylselenenyl chlorides with cycloalkenes proceeded as anti-addition affording adducts with trans-configuration [55,56,57,58]. The anti-addition was also observed in the reactions of 2-pyridinesulfenyl and selenenyl halides with isoeugenol, methyl isoeugenol, and trans-anethole affording products **6**, **8**, **11**–**13**, **16** with trans-configuration.

We attempted the reaction of 2-pyridinesulfenyl chloride with one representative of linear 1-alkenes: 1-heptene and observed the formation of two regioisomers **24** and **25** in a 9:2 ratio (Scheme 5). The major product **24** was derived from anti-Markovnikov addition to the double bond. Thus, like 1-alkenes, allylbenzene reacted with 2-pyridinesulfenyl chloride affording heterocycle **23** derived from anti-Markovnikov addition to the double bond (Scheme 5).

The structural assignments of the synthesized compounds were made using ^1^H and ^13^C-NMR spectroscopy including two-dimensional and NOESY experiments and confirmed by elemental analysis. The products of opposite regiochemistry have characteristic signals of the carbon atoms bonded with charged nitrogen (N^+^) in ^13^C-NMR spectra: CH_2_N^+^ (63–67 ppm) and (Ar)CHN^+^ (75–84 ppm). The values of proton spin-spin coupling constant (^3^*J*_H-H_) in the (Me)CH-CH(Ar)N fragment of the dihydrothiazole cycle correspond to trans-configuration of these protons. 

## 3. Experimental Section

### 3.1. General Information

^1^H (400.1 MHz) and ^13^C (100.6 MHz) NMR spectra were recorded on a Bruker DPX-400 spectrometer (Bruker BioSpin GmbH, Rheinstetten, Germany) in 5–10% solution in D_2_O or DMSO-*d*6 or CDCl_3_. ^1^H and ^13^C chemical shifts (δ) are reported in parts per million (ppm), relative to tetramethylsilane (external) or to the residual solvent peaks of DMSO-d6 (δ = 2.50 and 39.52 ppm in ^1^H- and ^13^C-NMR, respectively) or CDCl_3_ (δ = 7.26 and 77.16 ppm in ^1^H- and ^13^C-NMR, respectively). Spectral characteristics of compounds **19** and **20** are described elsewhere [32,35]. Elemental analysis was performed on a Thermo Scientific FLASH 2000 Organic Elemental Analyzer (Thermo Fisher Scientific Inc., Milan, Italy). Melting points were determined on a Kofler Hot-Stage Microscope PolyTherm A apparatus (Wagner & Munz GmbH, München, Germany). Absolute solvents were used in the reactions.

### 3.2. Synthesis of Compounds ***1**–**18***, ***21**–**24***

*trans-3-(4-Hydroxy-3-methoxyphenyl)-2-methyl-2H,3H-[1,3]thiazolo[3,2-а]pyridin-4-ium chloride* (**1**). A solution of sulfuryl chloride (0.135 g, 1 mmol) in chloroform (10 mL) was added dropwise to a solution of di(2-pyridine) disulfide (0.218 g, 1 mmol) in chloroform (10 mL) and the mixture was stirred for 10 min at room temperature. A solution of isoeugenol (0.328 g, 2 mmol) in chloroform (10 mL) was added dropwise and the reaction mixture stirred for 4 h at room temperature and 8 h at reflux temperature. On cooling the formed precipitate was filtered off and dried in vacuum giving the product (0.527 g, 85% yield) as a white powder, mp 235–237 °C. ^1^H-NMR (400 MHz, D_2_O): δ 1.56 (d, *J* = 6.7 Hz, 3H, CH_3_), 3.86 (s, 3H, OCH_3_), 4.58 (dq, *J* = 11.3, 6.7 Hz, 1H, SCH), 5.81 (d, *J* = 11.3 Hz, 1H, NCH), 6.99–7.04 (m, 2H, Ar), 7.14 (s, 1H, Ar), 7.55–7.59 (m, 1H, Py), 7.97–7.99 (m, 1H, Py), 8.10–8.12 (m, 1H, Py), 8.25–8.29 (m, 1H, Py). ^13^C-NMR (101 MHz, D_2_O): δ 17.58 (CH_3_), 51.37 (SCH), 57.94 (OCH_3_), 83.56 (NCH), 114.01 (Ar), 118.12 (Ar), 124.64 (Py), 125.39 (Py), 126.52(Ar), 143.22 (Py), 146.75 (Py), 149.11 (CO, Ar), 150.45 (CO, Ar), 161.81 (Py). Anal. Calcd for C_15_H_16_NClO_2_S: C 58.15, H 5.21, Cl 11.44, N 4.52, S 10.35. Found: C 57.91, H 5.07, Cl 11.63, N 4.34, S 10.13.

*2-[(4-Hydroxy-3-methoxyphenyl)methyl]-2H,3H-thiazolo[3,2-а]pyridin-4-ium chloride* (**2**) was obtained in 74% yield as a yellowish powder, mp 208–210 °C, from 2-pyridinesulfenyl chloride and eugenol under similar conditions as synthesis of compound **1**. ^1^H-NMR (400 MHz, DMSO-*d*_6_): δ 3.03 (qd, *J* = 13.9, 7.2 Hz, 2H, CH_2_), 3.75 (s, 3H, ОCH_3_), 4.60–4.76 (m, 1H, SCH,), 5.10 (qd, *J =* 13.6, 6.5 Hz, 2H, NCH_2_), 6.68 (d, *J* = 8.0 Hz, 1H, Ar), 6.74 (d, *J* = 8.0 Hz, 1H, Ar), 6.89 (s, 1H, Ar), 7.69–7.73 (m, 1H, Py), 8.09–8.11 (m, 1H, Py), 8.28–8.31 (m, 1H, Py), 8.98–8.99 (m, 1H, Py). ^13^C-NMR (101 MHz, DMSO-*d*_6_): δ 38.51 (CH_2_), 48.32 (SCH), 55.65 (ОCH_3_), 63.31 (NCH_2_), 113.33 (Ar), 115.35 (Ar), 121.44 (Py), 122.40 (Py), 123.10 (Py), 127.52 (Ar), 142.91 (Ar), 144.40 (Py), 145.63 (СОH, Ar), 147.48 (СОСH_3_, Ar), 158.61 (NCS, Py). Anal. Calcd for C_15_H_16_NClO_2_S: С 58.15, H 5.21, Cl 11.44, N 4.52, S 10.35. Found: С 57.96, H 5.40, Cl 11.67, N 4.72, S 10.57.

*trans-3-(4-Hydroxy-3-methoxyphenyl)-2-methyl-2H,3H-selenazolo[3,2-а]pyridine-4-ium chloride* (**3**) was obtained in 73% yield from 2-pyridineselenenyl chloride and isoeugenol as a yellowish powder, mp 230–232 °C under similar conditions as synthesis of compound **1**. ^1^H-NMR (400 MHz, D_2_O): δ 1.62 (d, *J* = 6.7 Hz, 3H, CH_3_), 3.85 (s, 3H, OCH_3_), 4.70 – 4.62 (m, 1H, SeCH), 5.83 (d, *J* = 10.5 Hz, 1H, NCH), 6.93 (d, *J* = 8.2 Hz, 1H, Ar), 7.00 (d, *J* = 8.2 Hz, 1H, Ar), 7.10 (s, 1H, Ar), 7.57-7.61 (m, 1H, Py), 8.10 – 8.20 (m, 3H, Py). ^13^C-NMR (101 MHz, D_2_O): δ 16.26 (CH_3_), 44.81 (SeCH), 55.58 (OCH_3_), 83.73 (NCH), 111.53 (Ar), 115.74 (Ar), 121.97 (Py), 122.95 (Py), 125.04 (Py), 127.13 (Ar), 142.39 (Ar), 143.59 (Py), 146.60 (СОCH_3_, Ar), 148.10 (СОH, Ar), 158.03 (NCSe, Py). Anal. Calcd for C_15_H_16_NClO_2_Se: С 50.51; H 4.52; N 3.93, Cl 9.94, Se 22.14. Found: С 50.62; H 4.41; N 3.81, Cl 9.71, Se 22.37.

*2-[(4-Hydroxy-3-methoxyphenyl)methyl]-2H,3H-selenazolo[3,2-а]pyridin-4-ium chloride* (**4**) was obtained in 70% yield from 2-pyridineselenenyl chloride and eugenol as a yellowish powder, mp 206–208 °C under similar conditions as synthesis of compound **1**. ^1^H-NMR (400 MHz, D_2_O): δ 3.00-3.03 (m, 2H, CH_2_), 3.69 (s, 3H, ОCH_3_), 4.56 (s, 1H, SeCH,), 4.99 (s, 2H, NCH_2_), 6.61 (s, 2H, Ar), 6.78 (s, 1H, Ar), 7.46–7.48 (m, 1H, Py), 7.79–7.81 (m, 1H, Py), 7.91–7.95 (m, 1H, Py), 8.44–8.45 (m, 1H, Py). ^13^C-NMR (101 MHz, D_2_O): δ 38.72 (CH_2_), 45.19 (SeCH), 55.68 (ОСH_3_), 66.48 (NCH_2_), 112.96 (Ar), 115.11 (Ar), 121.88 (Py), 122.90 (Py), 126.94 (Py), 131.50 (Ar), 142.42 (Ar), 143.07 (Py), 144.23 (СОH, Ar), 145.46 (СОСH_3_, Ar), 158.72 (NCSe, Py). Anal. Calcd for C_15_H_16_NClO_2_Se: С 50.51; H 4.52; N 3.93, Cl 9.94, Se 22.14. Found: С 50.78; H 4.35; N 4.12, Cl 10.13, Se 21.93.

*2-[(3,4-dimethoxyphenyl)methyl]-2H,3H-[1,3]thiazolo[3,2-а]pyridin-4-ium chloride* (**5**). A solution of sulfuryl chloride (0.068 g, 0.5 mmol) in methylene chloride (7 mL) was added dropwise to a solution of di(2-pyridine) disulfide (0.109 g, 0.5 mmol) in methylene chloride (7 mL) and the mixture was stirred for 10 min at room temperature. A solution of methyl eugenol (0.178 g, 1 mmol) in methylene chloride (7 mL) was added dropwise and the reaction mixture was stirred for 20 h at room temperature. The solvent was removed by rotary evaporator (RE-52AA, Xi’an Heb Biotechnology Co., Xi’an, China) and the residue was dried in vacuum giving the product (0.324 g, quantitative yield) as a light yellow oil. ^1^H-NMR (400 MHz, D_2_O): δ 3.00–3.10 (m, 2H, CH_2_), 3.73 (s, 3H, ОCH_3_), 3.77 (s, 3H, ОCH_3_), 4.58 (t, *J* = 5.1 Hz, 1H, SCH), 5.03 (qd, *J =* 13.6, 5.7 Hz, 2H, NCH_2_), 6.80 (s, 2H, Ar), 6.87 (s, 1H, Ar), 7.47–7.50 (m, 1H, Py), 8.72–8.74 (m, 1H, Py), 8.06–8.10 (m, 1H, Py), 8.47–8.48 (m, 1H, Py). ^13^C-NMR (101 MHz, D_2_O): δ 38.63 (CH_2_), 48.26 (SCH), 55.65 (ОСH_3_), 63.83 (NCH_2_), 111.75 (Ar), 112.66 (Ar), 122.15 (Py), 122.47 (Py), 123.14 (Py), 128.87 (Ar), 141.51 (Ar), 144.24 (Py), 147.34 (СОСH_3_, Ar), 147.77 (СОСH_3_, Ar), 159.44 (NCS, C_5_H_4_N). Anal. Calcd for C_16_H_18_NClO_2_S: С 59.34, H 5.60, Cl 10.95, N 4.33, S 9.90. Found: С 59.09, H 5.78, Cl 11.14, N 4.52, S 10.05.

*trans-3-(3,4-dimethoxyphenyl)-2-methyl-2H,3H-[1,3]thiazolo[3,2-а]pyridin-4-ium chloride* (**6**). A solution of sulfuryl chloride (0.068 g, 0.5 mmol) in chloroform (7 mL) was added dropwise to a solution of di(2-pyridine) disulfide (0.109 g, 0.5 mmol) in chloroform (7 mL) and the mixture was stirred for 10 min at room temperature. A solution of methyl isoeugenol (0.178 g, 1 mmol) in chloroform (7 mL) was added dropwise and the reaction mixture stirred for 1 h at room temperature and 5 h at reflux temperature. The solvent was removed by rotary evaporator and the residue was dried in vacuum giving the product (0.324 g, quantitative yield) as a light yellow oil. ^1^H-NMR (400 MHz, D_2_O): δ 1.55 (d, *J* = 6.7 Hz, 3H, CH_3_), 3.82 (s, 3H, ОCH_3_), 3.87 (s, 3H, ОCH_3_), 4.53 (dq, *J =* 11.3, 6.7 Hz, 1H, SCH), 5.82 (d, *J =* 11.3 Hz, 1H, NCH), 7.08–7.14 (m, 3H, Ar), 7.54–7.58 (m, 1H, Py), 7.96–7.98 (m, 1H, Py), 8.09–8.11 (m, 1H, Py), 8.24–8.28 (m, 1H, Py). ^13^C-NMR (101 MHz, D_2_O): δ 15.60 (CH_3_), 49.37 (SCH), 55.70 (ОСH_3_), 81.47 (NCH), 111.00 (Ar), 112.15 (Ar), 122.72 (Py), 123.41 (Py), 125.02 (Ar), 141.24 (Ar), 144.78 (Py), 149.17 (СОCH_3_, Ar), 150.02 (СОСH_3_, Ar), 159.88 (NCS, Py), Anal. Calcd for C_16_H_18_NClO_2_S: С 59.34, H 5.60, Cl 10.95, N 4.33, S 9.90. Found: С 59.62, H 5.81, Cl 11.16, N 4.51; S 10.12. 

*2-[(3,4-Dimethoxyphenyl)methyl]-2H,3H-[1,3]selenazolo[3,2-а]pyridin-4-ium chloride* (**7**) was obtained as a light yellow oil in quantitative yield from 2-pyridineselenenyl chloride and methyl eugenol under similar conditions as synthesis of compound **5**. ^1^H-NMR (400 MHz, D_2_O): δ 3.15 (s, 2H, CH_2_), 3.75 (d, *J* = 12.3 Hz, 6H, ОCH_3_), 4.66 (s, 1H, SeCH), 5.06–5.15 (m, 2H, NCH_2_), 6.82 (s, 2H, Ar), 6.90 (s, 1H, Ar), 7.50 (s, 1H, Py), 7.87–7.89 (m, 1H, Py), 7.96–7.98 (m, 1H, Py), 8.50 (s, 1H, Py). ^13^C-NMR (101 MHz, D_2_O): δ 38.85 (CH_2_), 44.94 (SeCH), 55.50 (ОСH_3_), 66.58 (NCH_2_), 111.66 (Ar), 112.49 (Ar), 121.93 (Py), 122.89 (Py), 126.96 (Ar), 142.46 (Ar), 143.07 (Py), 146.09 (СОСH_3_, С_6_H_3_), 147.12 (СОСH_3_, Ar), 157.90 (NCSe, Py). Anal. Calcd for C_16_H_18_NClO_2_Se: С 51.84; H 4.89; N 3.78, Cl 9.56, Se 21.30. Found: С 52.56; H 5.16; N 4.25, Cl 9.34, Se 21.57.

*trans-3-(3,4-Dimethoxyphenyl)-2-methyl-2H,3H-[1,3]selenazolo[3,2-а]pyridin-4-ium chloride* (**8**) was obtained as a light yellow oil in quantitative yield from 2-pyridineselenenyl chloride and methyl isoeugenol under similar conditions as synthesis of compound **6**. ^1^H-NMR (400 MHz, D_2_O): δ 1.55 (d, *J* = 6.8 Hz, 3H, CH_3_), 3.84 (s, 3H, ОCH_3_), 3.89 (s, 3H, ОCH_3_), 4.67–4.71 (m, 1H, SeCH), 5.87 (d, *J =* 10.4 Hz, 1H, NCH), 7.04–7.06 m (1H, Ar), 7.12 (dd, *J =* 14.4, 5.1 Hz, 2H, Ar), 7.58–7.62 (m, 1H, Py), 8.11–8.21 (m, 3H, Py). ^13^C-NMR (101 MHz, D_2_O): δ 16.81 (CH_3_), 45.26 (SeCH), 55.84 (ОСH_3_), 84.07 (NCH), 111.04 (Ar), 112.30 (Ar), 122.44 (Py), 123.45 (Py), 126.03 (Ar), 127.61 (Ar), 142.24 (Py), 144.09 (Py), 149.29 (СОH, Ar), 150.00 (СОСH_3_, Ar), 158.56 (NCS, Py). Anal. Calcd for C_16_H_18_NClO_2_Se: С 51.84, H 4.89, N 3.78, Cl 9.56, Se 21.30. Found: С 52.00, H 5.02, N 3.91, Cl 9.73, Se 21.03. 

*2-[[4-(Acethyloxy)-3-methoxybenzyl]-2H,3H-[1,3]thiazolo[3,2-а]pyridin-4-ium chloride* (**9**) was obtained as a light yellow oil in quantitative yield from 2-pyridinesulfenyl chloride and acetyleugenol under similar conditions as synthesis of compound **5**. ^1^H-NMR (400 MHz, D_2_O): δ 2.32 (s, 3H, CH_3_), 3.14–3.23 (m, 2H, CH_2_), 3.81 (s, 3H, ОCH_3_), 4.64–4.70 (m, 1H, SCH), 5.06–5.16 (m, 2H, NCH_2_,), 6.95 (dd, *J* = 8.1, 1.8 Hz, 1H, Ar), 7.00 (d, *J* = 8.1 Hz, 1H, Ar), 7.09 (d, *J* = 1.8 Hz, 1H, Ar), 7.53–7.55 (m, 1H, Py), 7.80–7.82 (m, 1H, Py), 8.11–8.16 (m, 1H, Py), 8.51–8.53 (m, 1H, Py). ^13^C-NMR (101 MHz, D_2_O): δ 19.59 (СH_3_), 38.57 (CH_2_), 47.37 (SCH), 55.71 (ОСH_3_), 63.47 (NCH_2_), 113.74 (Ar), 121.89 (Ar), 122.26 (Py), 122.33 (Py), 122.84 (Py), 135.26 (Ar), 137.84 (Ar), 141.11 (Ar), 144.06 (Py), 149.73 (СОCH_3_, Ar), 159.00 (NCS, Py), 172.30 (СОOCH_3,_ Ar). Anal. Calcd for C_17_H_18_NClO_3_S: С 58.03, H 5.16, N 3.98, Cl 10.08, S 9.11. Found: С 58.25, H 5.34, N 4.15, Cl 10.36, S 9.38.

*2-[[4-(Acetyloxy)-3-methoxybenzyl]-2H,3H-[1,3]selenazolo[3,2-а]pyridin-4-ium chloride* (**10**) was obtained as a light yellow oil in quantitative yield from 2-pyridineselenenyl chloride and acetyleugenol under similar conditions as synthesis of compound **6**. ^1^H-NMR (400 MHz, D_2_O): δ 2.31 (s, 3H, CH_3_), 3.26 (s, 2H, CH_2_), 3.80 (s, 3H, ОCH_3_), 4.70 (s, 1H, SeCH), 5.09–5.21 (m, 2H, NCH_2_,), 6.94-6.99 (m, 2H, Ar), 7.08 (s, 1H, Ar), 7.56–7.59 (m, 1H, Py), 7.93–7.95 (m, 1H, Py), 8.02–8.04 (m, 1H, Py), 8.58–8.59 (m, 1H, Py). ^13^C-NMR (101 MHz, D_2_O): δ 19.84 (СH_3_), 39.15 (CH_2_), 44.46 (SeCH), 55.96 (ОСH_3_), 66.62 (NCH_2_), 113.86 (Ar), 122.00 (Ar), 122.54 (Py), 123.16 (Py), 127.09 (Py), 136.38 (Ar), 138.02 (Ar), 142.48 (Ar), 143.37 (Py), 149.92 (СОCH_3_, Ar), 157.76 (NCSe, Py), 172.59 (СОOCH_3_). Anal. Calcd for C_17_H_18_NClO_3_Se: С 51.21, H 4.55, N 3.51, Cl 8.89, Se 19.80. Found: С 51.42, H 4.72, N 3.68, Cl 9.05, Se 20.07.

*trans-3-(4-Methoxyphenyl)-2-methyl-2H,3H-thiazolo[3,2-а]pyridin-4-ium chloride* (**11**) was obtained in quantitative yield from 2-pyridinesulfenyl chloride and trans-anethole as a yellowish powder, mp 140–142 °C under similar conditions as the synthesis of compound **5**. ^1^H-NMR (400 MHz, D_2_O): δ 1.54–1.56 (m, 3H, CH_3_), 3.86 (s, 3H, ОCH_3_), 4.52 (dd, *J* = 10.6, 6.6 Hz, 1H, SCH), 5.88 (dd, *J* = 10.6, 2.9 Hz, 1H, NCH), 7.10–7.13 (m, 2H, Ar), 7.44–7.47 (m, 2H, Ar), 7.55–7.58 (m, 1H, Py), 7.98–7.99 (m, 1H, Py), 8.11–8.12 (m, 1H, Py), 8.25–8.28 (m, 1H, Py). ^13^C-NMR (101 MHz, D_2_O): δ 16.06 (СH_3_), 49.58 (SCH), 55.60 (ОСH_3_), 81.25 (NCH), 115.34 (Ar), 122.85 (Py), 123.55 (Py), 125.11 (Ar), 130.36 (Ar), 141.37 (Py), 144.89 (Py), 159.90 (NCS, Py), 160.76 (СОСH_3_, Ar). Anal. Calcd for C_15_H_16_NclOS: С 61.32, H 5.49, N 4.77, Cl 12.07, S 10.91. Found: С 61.53, H 5.58, N 4.98, Cl 11.85, S 11.04. 

*trans-3-(4-Methoxyphenyl)-2-methyl-2H,3H-selenazolo[3,2-а]pyridin-4-ium chloride* (**12**). A solution of sulfuryl chloride (0.122 g, 0.9 mmol) in chloroform (10 mL) was added dropwise to a solution of di(2-pyridine) diselenide (0.28 g, 0.9 mmol) in chloroform (20 mL) and the mixture was stirred for 20 min at room temperature. A solution of trans-anethole (0.266 g, 1.8 mmol) in chloroform (10 mL) was added dropwise and the reaction mixture stirred for 1 h at room temperature and 4 h at reflux temperature. The solvent was removed by rotary evaporator and the residue was dried in vacuum giving the product (0.613 g) in quantitative yield as a yellowish powder, mp 141–143 °C. ^1^H-NMR (400 MHz, D_2_O): δ 1.67 (d, *J* = 6.6 Hz, 3H, CH_3_,), 3.83 (s, 3H, ОCH_3_), 4.60 (qd, *J* = 13.4, 6.6 Hz, 1H, SeCH), 5.97 (d, 1H, *J* = 9.4 Hz, NCH), 7.12 (dd, *J* = 9.0, 2.5 Hz, 2H, Ar), 7.41 (dd, *J* = 9.0, 2.5 Hz, 2H, Ar), 7.63–7.66 (m, 1H, Py), 8.16–8.26 (m, 3H, Py). ^13^C-NMR (101 MHz, D_2_O): δ 17.54 (СH_3_), 45.62 (SeCH), 55.69 (ОСH_3_), 83.78 (NCH), 115.37 (Ar), 123.60 (Py), 126.10 (Ar), 127.76 (Py), 130.05 (Ar), 142.96 (Py), 144.21 (Py), 158.58 (NCSe, Py), 160.61 (СОСH_3_, Ar). Anal. Calcd for C_15_H_16_NclOSe: С 52.88, H 4.73, N 4.11, Cl 10.41, Se 23.18. Found: С 53.11, H 4.93, N 4.36, Cl 10.60, Se 23.40.

*trans-3-(4-Methoxyphenyl)-2-methyl-2H,3H-thiazolo[3,2-а]pyridin-4-ium bromide* (**13**). A solution of bromine (0.122 g, 0.76 mmol) in methylene chloride (8 mL) was added dropwise to a solution of di(2-pyridine) disulfide (0.168 g, 0.76 mmol) in methylene chloride (8 mL) and the mixture was stirred for 30 min at room temperature. A solution of trans-anethole (0.225 g, 1.52 mmol) in methylene chloride (8 mL) was added dropwise and the reaction mixture stirred for 24 h at room temperature. The mixture was filtered and the solvent was removed by rotary evaporator. The residue was dried in vacuum giving the product (0.411 g, 80% yield) as a light yellow oil. ^1^H-NMR (400 MHz, D_2_O): δ 1.57–1.58 (m, 3H, CH_3_), 3.88 (s, 3H, ОCH_3_), 4.51–4.58 (m, 1H, SCH), 5.91 (d, *J* = 10.6 Hz, 1H, NCH), 7.13–7.15 (m, 2H, Ar), 7.47–7.49 (m, 2H, Ar), 7.58–7.60 (m, 1H, Py), 7.99–8.01 (m, 1H, Py), 8.12–8.14 (m, 1H, Py), 8.26–8.30 (m, 1H, Py). ^13^C-NMR (101 MHz, D_2_O): δ 15.98 (СH_3_), 49.50 (SCH), 55.54 (ОСH_3_), 81.15 (N^+^CH), 115.26 (С_6_H_4_), 122.77 (Py), 123.48 (Py), 125.00 (С_6_H_4_), 130.31 (С_6_H_4_), 141.29 (Py), 144.79 (Py), 159.80 (NCS, Py), 160.66 (СОСH_3_, С_6_H_4_). Anal. Calcd for C_15_H_16_NBrOS: С 53.26, H 4.77, N 4.14, Br 23.62, S 9.48. Found: С 53.58, H 4.90, N 4.32, Br 23.92, S 9.71.

*3-(4-Methylphenyl)-2H,3H-thiazolo[3,2-а]pyridin-4-ium bromide* (**14**) was obtained in 84% yield from 2-pyridinesulfenyl bromide and 4-methylstyrene as a yellowish powder, mp 199–201 °C under similar conditions as synthesis of compound **13**. ^1^H-NMR (400 MHz, D_2_O): δ 2.33 (s, 3H, CH_3_), 3.86 (dd, *J* = 11.8, 9.9 Hz, 1H, SCH_2_), 4.21 (dd, *J* = 11.8, 8.7 Hz, 1H, SCH_2_), 6.37 (t, *J =* 8.7 Hz, 1H, NCH), 7.32–7.38 (m, 4H, Ar), 7.56–7.59 (m, 1H, Py), 8.04–8.06 (m, 1H, Py), 8.15–8.17 (m, 1H, Py), 8.27–8.31 (m, 1H, Py). ^13^C-NMR (101 MHz, D_2_O): δ 21.38 (CH_3_), 37.63 (SCH_2_), 76.16 (NCH), 123.68 (Py), 124.31 (Py), 128.85 (Ar), 131.26 (Ar), 132.06 (Ar), 141.91 (Py), 142.02 (Ar), 145.54 (Py), 160.95 (SCN^+^, Py). Anal. Calcd for C_14_H_14_NBrS: С 54.55, H 4.58, N 4.54, Br 25.92, S 10.40. Found: С 54.68, H 4.62, N 4.78, Br 26.23, S 10.69.

*3-(4-Methylphenyl)-2H,3H-thiazolo[3,2-а]pyridin-4-ium chloride* (**15**) was obtained in quantitative yield from 2-pyridinesulfenyl chloride and 4-methylstyrene as a yellowish oil under similar conditions as synthesis of compound **5.** ^1^H-NMR (400 MHz, D_2_O): δ 2.30 (s, 3H, CH_3_), 3.78 – 3.88 (m, 1H), 4.14 (dd, *J* = 11.9, 8.0 Hz, 1H, SCH_2_), 6.29 (t, *J* = 8.9 Hz, 1H, NCH), 7.27–7.33 (m, 4H, Ar), 7.50–7.53 (m, 1H, Py), 7.97–7.99 (m, 1H, Py), 8.10–8.12 (m, 1H, Py), 8.21–8.25 (m, 1H, Py). ^13^C-NMR (101 MHz, D_2_O): δ 20.50 (CH_3_), 36.70 (SCH_2_), 75.49 (NCH), 122.88 (Py), 123.48 (Py), 128.05 (Ar), 130.47 (Ar), 131.32 (Ar), 141.16 (Py), 141.34 (Ar), 144.76 (Py), 160.26 (NCS, Py).Anal. Calcd for C_14_H_14_NClS: С 63.74, H 5.35, N 5.31, Cl 13.44. S 12.16. Found: С 63.94, H 5.49, N 5.51, Cl 13.17. S 11.97.

*trans-3-(4-Methoxyphenyl)-2-methyl-2H,3H-[1,3]selenazolo[3,2-а]pyridin-4-ium bromide* (**16**). A solution of bromine (0.051 g, 0.32 mmol) in chloroform (10 mL) was added dropwise to a solution of di(2-pyridine) diselenide (0.1 g, 0.32 mmol) in chloroform (10 mL) and the mixture was stirred for 20 min at room temperature. A solution of trans-anethole (0.095 g, 0.64 mmol) in chloroform (10 mL) was added dropwise and the reaction mixture stirred for 1 h at room temperature and 5 h at reflux temperature. The mixture was filtered and the solvent was removed by rotary evaporator. The residue was dried in vacuum giving the product (0.234 g, 95% yield) as a light orange oil. ^1^H-NMR (400 MHz, D_2_O): δ 1.62 (d, *J* = 6.5 Hz, 3H, CH_3_,), 3.85 (s, 3H, ОCH_3_), 4.57–4.61 (m, 1H, SeCH), 5.91 (d, 1H, *J* = 9.7 Hz, NCH), 7.10 (d, *J* = 8.3 Hz, 2H, Ar), 7.39 (d, *J* = 8.3 Hz, 2H, Ar), 7.59–7.60 (m, 1H, Py), 8.11–8.20 (m, 3H, Py). ^13^C-NMR (101 MHz, D_2_O): δ 17.19 (СH_3_), 45.48 (SeCH), 55.56 (ОСH_3_), 83.68 (NCH), 115.25 (Ar), 123.41 (Py), 125.84 (Ar), 127.61 (Py), 130.05 (Ar), 142.85 (Py), 144.02 (Py), 158.47 (NCSe, Py), 160.52 (СОСH_3_, Ar). Anal. Calcd for C_15_H_16_NbrOSe: С 46.78, H 4.19, N 3.64, Br 20.75, Se 20.50. Found: С 48.02, H 4.48, N 3.98, Br 21.03, Se 20.21.

*3-(4-Methylphenyl)-2H,3H-selenazolo[3,2-а]pyridin-4-ium chloride* (**17**) was obtained as a light yellow oil in quantitative yield from 2-pyridineselenenyl chloride and 4-methylstyrene under similar conditions as the synthesis of compound **16**. ^1^H-NMR (400 MHz, D_2_O) δ 2.30 (s, 1H, CH_3_), 3.81–3.86 (m, 1H, SeCH_2_), 4.09–4.14 (m, 1H, SeCH_2_), 6.28 (t, *J* = 8.4 Hz, 1H, NCH), 7.26–7.29 (m, 4H, Ar), 7.54 (ddd, *J* = 6.5, 2.4, 1.0 Hz, 1H, Py), 8.10–8.17 (m, 3H, Py). ^13^C-NMR (101 MHz, D_2_O) δ 19.82 (CH_3_), 30.21 (SeCH_2_), 77.27 (NCH), 122.79 (Py), 126.71 (Py), 127.28 (Ar), 129.78 (Ar), 131.31 (Ar), 140.49 (Ar), 141.93 (Py), 143.23 (Py), 157.93 (NCSe, Py). Anal. Calcd for C_14_H_14_NclSe: С 54.12, H 4.54, N 4.51, Cl 11.41, Se 25.42. Found: С 53.87, H 4.68, N 4.39, Cl 11.56, Se 25.69.

*3-Phenyl-2H,3H-selenazolo[3,2-a]pyridin-4-ium bromide* (**18**) was obtained as a light orange oil in 78% yield from 2-pyridineselenenyl bromide and styrene under similar conditions as the synthesis of compound **16**. ^1^H-NMR (400 MHz, DMSO-*d*_6_): δ 3.78 (dd, *J* = 10.9, 7.4 Hz, 1H, SCH_2_), 4.26 (dd, *J* = 10.9, 7.4 Hz, 1H, SCH_2_), 6.58 (t, *J* = 7.4 Hz, 1H, NCH), 7.38–7.40 (m, 2H, Ar), 7.48–7.50 (m, 3H, Ar), 7.71–7.75 (m, 1H, Py), 8.30–8.34 (m, 1H, Py), 8.42–8.44 (m, 1H, Py), 8.49–8.51 (m, 1H, Py). ^13^C-NMR (101 MHz, DMSO-*d*_6_): δ 32.13 (SCH_2_), 76.37 (NCH), 123.55 (Py), 127.44 (Py), 127.44 (Ar), 129.38 (Ar), 129.71 (Ar), 136.08 (Ar), 143.30 (Py), 143.95 (Py), 159.32 (NCS, Py). Anal. Calcd for C_13_H_12_BrNSe: С 45.77, H 3.55, N 4.11, Br 23.43, Se 23.15. Found: С 45.98, H 3.69, N 4.23, Br 23.65, Se 22.96.

*3-Phenyl-2H,3H-selenazolo[3,2-a]pyridin-4-ium chloride* (**19**) was obtained as a yellowish powder (mp 205–207 °C) in quantitative yield from 2-pyridineselenenyl chloride and styrene under similar conditions as synthesis of compound **5**. Spectral characteristics of compound **19** are described [32].

*3-Phenyl-2H,3H-[1,3]thiazolo[3,2-a]pyridin-4-ium chloride* (**20**) was obtained as a yellowish powder (mp 209–211 °C) in quantitative yield from 2-pyridinesulfenyl chloride and styrene under similar conditions as synthesis of compound **5.** Spectral characteristics of compound **20** are described [35].

*3-Phenyl-2H,3H-[1,3]thiazolo[3,2-a]pyridin-4-ium bromide* (**21**) was obtained as a light yellow oil in 90% yield from 2-pyridinesulfenyl bromide and styrene under similar conditions as the synthesis of compound **16**. ^1^H-NMR (400 MHz, D_2_O): δ 3.95 (dt, *J* = 23.7, 11.8 Hz, 1H, SCH_2_), 4.23 (dt, *J* = 16.4, 8.2 Hz, 1H, SCH_2_), 6.37–6.42 (m, 1H, NCH), 7.50–7.52 (m, 2H, Ar, Py), 7.56–7.59 (m, 4H, Ar), 8.02–8.04 (m, 1H, Py), 8.20–8.22 (m, 1H, Py), 8.26–8.30 (m, 1H, Py). ^13^C-NMR (101 MHz, D_2_O): δ 36.77 (SCH_2_), 75.75 (NCH), 122.87 (Py), 123.49 (Py), 128.22 (Ar), 129.89 (Ar), 130.76 (Ar), 134.27 (Ar), 141.35 (Py), 144.76 (Py), 156.45 (NCS, Py). Anal. Calcd for C_13_H_12_BrNS: С 53.07, H 4.11, N 4.76, Br 27.16, S 10.90. Found: С 53.48, H 4.29, N 5.01, Br 27.36, S 11.12.

*3-Methyl-3-phenyl-2H,3H-thiazolo[3,2-a]pyridin-4-ium chloride* (**22**) was obtained as a light yellowish oil in 81% yield from 2-pyridinesulfenyl chloride and α-methylstyrene under similar conditions as synthesis of compound **16**. ^1^H-NMR (400 MHz, D_2_O): δ 2.20 (s, 3H, CH_3_), 4.00 (d, *J* = 12.2 Hz, 1H, SCH_2_), 4.13 (d, *J* = 12.2 Hz, 1H, SCH_2_), 7.36–7.39 (m, 2H, Ar), 7.51–7.52 (m, 3H, Ar), 7.64–7.67 (m, 1H, Py), 8.03–8.05 (m, 1H, Py), 8.29–8.32 (m, 1H, Py). ^13^C-NMR (101 MHz, D_2_O): δ 23.41 (CH_3_), 43.38 (SCH_2_), 81.28 (NC), 123.00 (Py), 123.33 (Py), 125.98 (Ar), 129.04 (Ar), 129.51 (Ar), 138.13 (Ar), 140.04 (Py), 144.27 (Py), 159.57 (NCS, Py). Anal. Calcd for C_14_H_14_NClS: С 63.74, H 5.35, N 5.31, Cl 13.44. S 12.16. Found: С 63.52, H 5.13, N 5.53, Cl 13.64. S 11.92.

*2-(Phenylmethyl)-2H,3H-thiazolo[3,2-а]pyridine-4-ium chloride* (**23**) was obtained in quantitative yield as a light yellow oil from 2-pyridinesulfenyl bromide and allylbenzene under similar conditions as the synthesis of compound **13** (but 60 h stirring at room temperature)**.** ^1^H-NMR (400 MHz, D_2_O): δ 3.17 (qd, *J* = 14.2, 6.7 Hz, 2H, CH_2_), 4.60–4.66 (m, 1H, SCH,), 5.06 (qd, *J* = 13.5, 5.9 Hz, 2H, NCH_2_), 7.25-7.31 (m, 5H, Ar), 7.49–7.52 (m, 1H, Py), 7.77–7.79 (m, 1H, Py), 8.08–8.12 (m, 1H, Py), 8.50–8.52 (m, 1H, Py).^13^C-NMR (101 MHz, D_2_O): δ 38.57 (CH_2_), 47.55 (SCH), 63.39 (NCH_2_), 122.07 (Py), 122.83 (Py), 123.47 (Py), 127.09 (Ar), 127.83 (Ar),128.26 (Ar), 128.83 (Ar), 143.90 (Py), 158.93 (NCS, Py). Anal. Calcd for C_14_H_14_NClS: С 63.74, H 5.35, N 5.31, Cl 13.44. S 12.16. Found: С 63.98, H 5.53, N 5.17, Cl 13.21. S 12.37.

*2-Pentyl-2H,3H-thiazolo[3,2-а]pyridin-4-ium chloride* (**24**) was obtained in 80% yield (in the mixture with compound **25**, a ratio **24**/**25** = 9:2) from 2-pyridinesulfenyl chloride and 1-heptene under similar conditions as synthesis of compound **13**. Compound **24** was characterized in the mixture with compound **25** (see Supplementary Materials for the ^1^H and ^13^C-NMR spectra pictures). ^1^H-NMR (400 MHz, CDCl_3_): δ 0.66–0.70 (m, 3H, CH_3_), 1.11–1.12 (m, 4H, CH_2_), 1.25–1.26 (m, 2H, CH_2_), 1.38 (dt, *J* = 16.2, 8.7 Hz, 1H, CH_2_), 1.77–1.86 (m, 1H, CH_2_), 4.23–4.30 (m, 1H, SCH), 5.14 (dd, *J* = 13.7, 7.2 Hz, 1H, CH_2_N), 5.27 (dd, *J* = 13.7, 7.5 Hz, 1H, CH_2_N), 7.52–7.56 (m, 1H, Py), 7.81–7.84 (m, 1H, Py), 8.16–8.20 (m, 1H, Py), 9.64–9.66 (m, 1H, Py). ^13^C-NMR (101 MHz, CDCl_3_): δ 13.70 (CH_3_), 22.07 (CH_2_), 27.02 (CH_2_), 30.87 (CH_2_), 33.42 (CH_2_), 48.80 (SCH), 64.79 (NCH_2_), 122.71 (Py), 123.03 (Py), 143.95 (Py), 144.65 (Py), 159.03 (NCS, Py).

## 4. Conclusions

Both eugenol and isoeugenol derivatives reacted with 2-pyridinesulfenyl and 2-pyridineselenenyl halides in a regioselective mode affording products with the opposite regiochemistry with respect to the location of aryl-containing substituents. Synthesis of new ensembles of 2*H*,3*H*-[1,3]thia- and -selenazolo[3,2-*a*]pyridin-4-ium heterocycles **1**–**18** and **21**–**24** in up to quantitative yields has been developed by annulation reactions of 2-pyridinechalcogenyl chlorides with natural compounds (eugenol, isoeugenol, methyl eugenol, methyl isoeugenol, trans-anethole) and their structural analogs. The obtained condensed heterocycles are novel water-soluble functionalized compounds with promising biological activity. 

First studies on the influence of the substrate structure and the nature of halogen and chalcogen on the product yields in the reactions of 2-pyridinesulfenyl and 2-pyridineselenenyl halides with alkenes were carried out. The introduction of methyl substituent at β-position of the double bond of styrene as well as to the position 4 of the benzene ring has little influence on the yields of products in annulation reactions. However, the introduction of methyl substituent at α-position of the double bond of styrene has negative effect on the annulation process. The 2-pyridinesulfenyl and 2-pyridineselenenyl chlorides are more efficient compared to corresponding bromides and the annulation reactions of 2-pyridinechalcogenyl chlorides usually afforded the desired products in higher (mostly quantitative) yields. Regarding the influence of the chalcogen nature, 2-pyridinesulfenyl and 2-pyridineselenenyl chlorides exhibit close reactivity. 

## Supplementary Materials

The following are available online https://www.mdpi.com/1420-3049/25/2/376/s1, examples of ^1^H- and ^13^C-NMR spectra of the obtained compounds.
