# Supplementary material for: Natural Compounds and Their Structural Analogs in Regio- and Stereoselective Synthesis of New Families of Water-Soluble 2H,3H-[1,3]thia- and -Selenazolo[3,2-a]pyridin-4-ium Heterocycles by Annulation Reactions
Source: Molecules. 2020 Jan 16;25(2):376. doi: 10.3390/molecules25020376 (PMC7024257; doi:10.3390/molecules25020376)
Supplement: Supplementary file 1 [file molecules-25-00376-s001.pdf]

Supporting information

Natural compounds and their structural analogs in regio- and stereoselective synthesis of new families of water-soluble 2**H**,3**H**-[1,3]thia- and -selenazolo[3,2-**a**]pyridin-4-ium heterocycles by annulation reactions

Vladimir A. Potapov[1,*], Roman S. Ishigeev[1], Irina V. Shkuchenko[1,2], Sergey V. Zinchenko[1] and Svetlana V. Amosova

[1] A. E. Favorsky Irkutsk Institute of Chemistry, Siberian Division of The Russian Academy of Sciences, 1 Favorsky Str., Irkutsk 664033, Russian Federation; v.a.potapov@mail.ru

[2] Irkutsk State University, Russia, Irkutsk 664003, 1 Karl Marx Str.

Table of Contents

Experimental (General Information) **2**

Examples of ^{1}H and ^{13}C-NMR Spectra **3-18**

Experimental (General Information)

^{1}H (400.1 MHz) and ^{13}C (100.6 MHz) NMR spectra were recorded on a Bruker DPX-400 spectrometer in 5-10% solution in D_2O or DMSO-*d*6 or $CDCl_3$. ^{1}H and ^{13}C chemical shifts (δ) are reported in parts per million (ppm), relative to tetramethylsilane (external) or to the residual solvent peaks of DMSO-d6 (δ = 2.50 and 39.52 ppm in ^{1}H- and ^{13}C-NMR, respectively) or $CDCl_3$ (δ = 7.26 and 77.16 ppm in ^{1}H- and ^{13}C-NMR, respectively).

Examples of ^{1}H and ^{13}C-NMR spectra

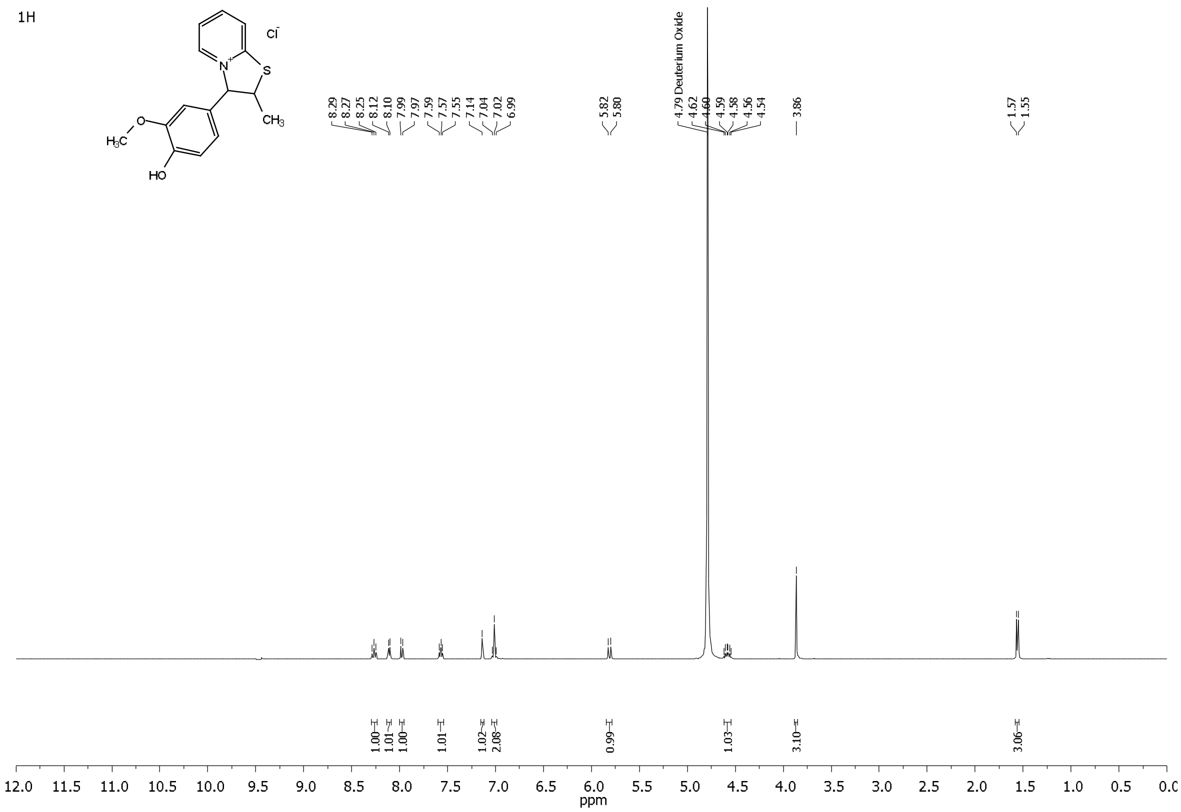

^{1}H-NMR (D_2O) spectrum of compound 1

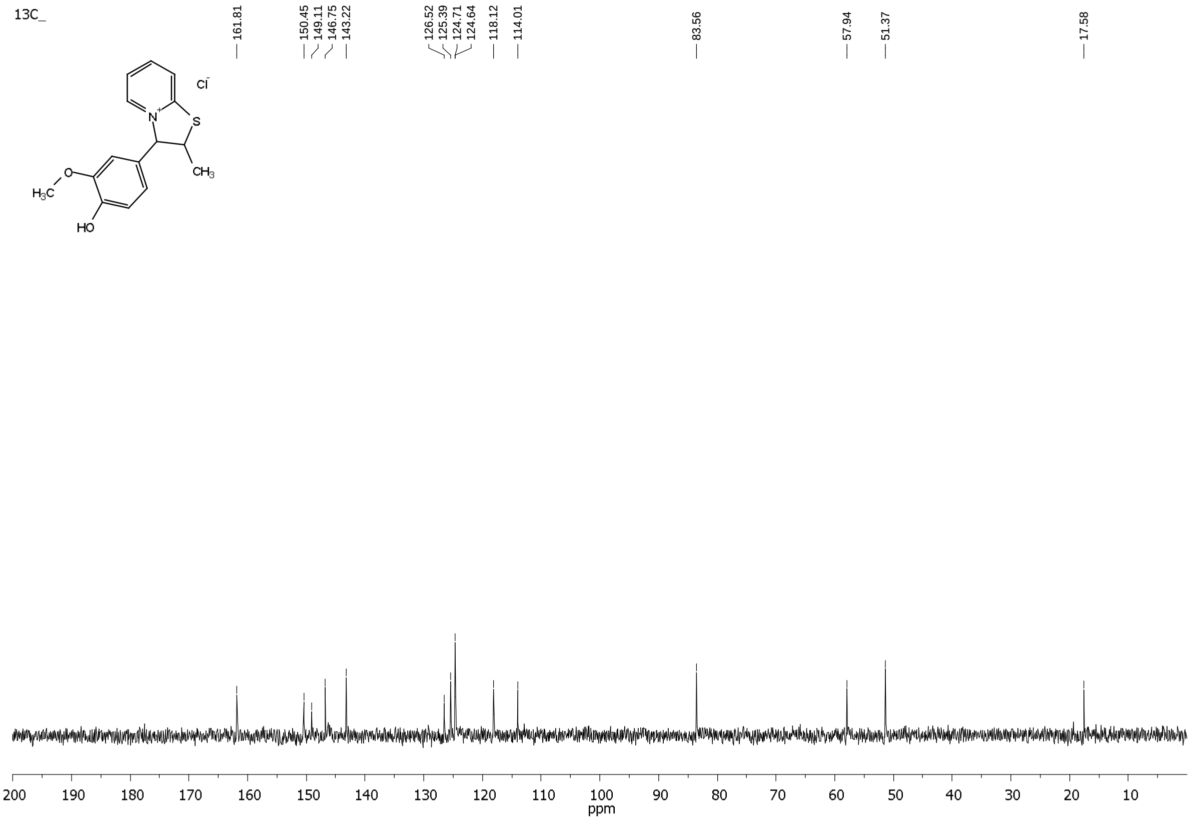

^{13}C-NMR (D_2O) spectrum of compound 1

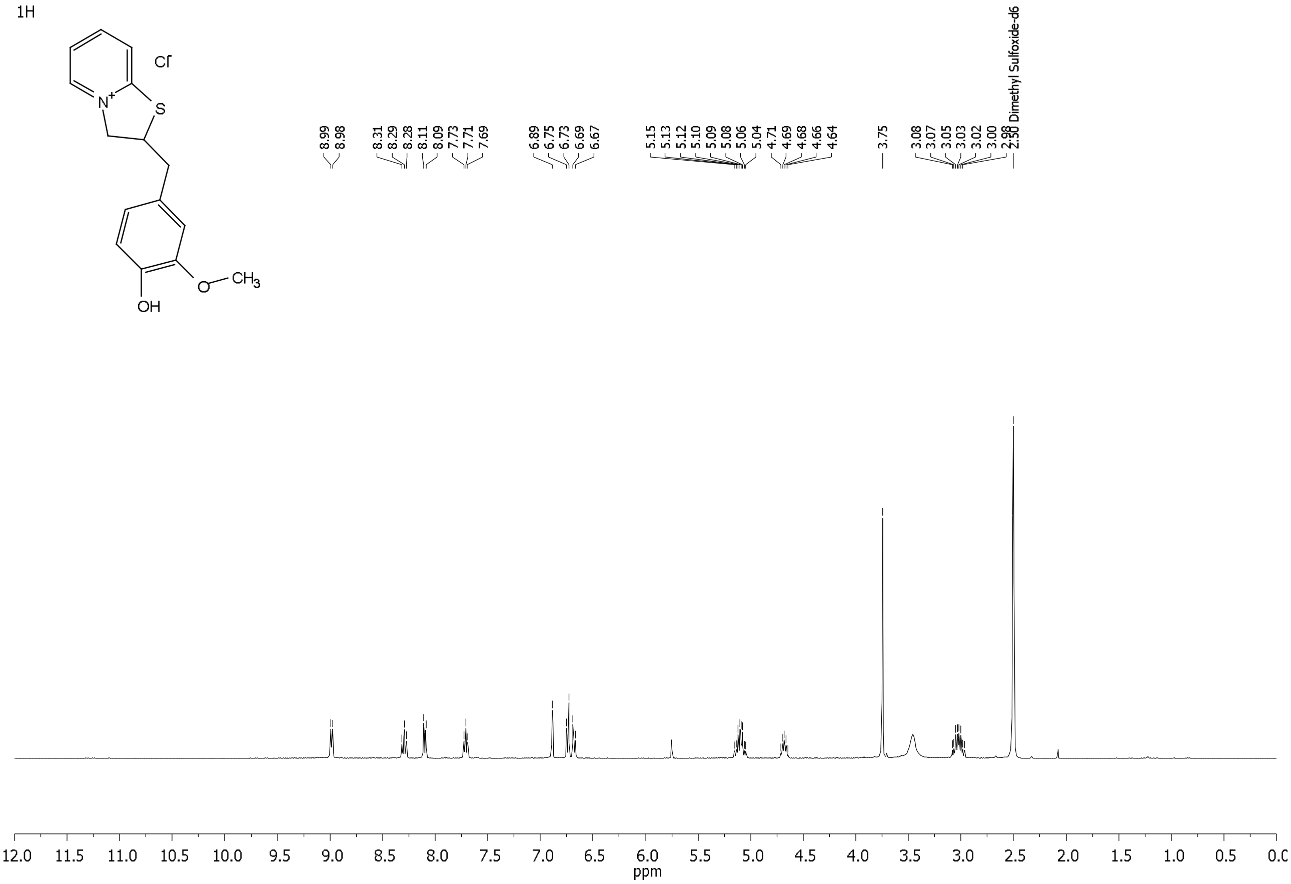

^{1}H-NMR (DMSO-d_6) spectrum of compound 2

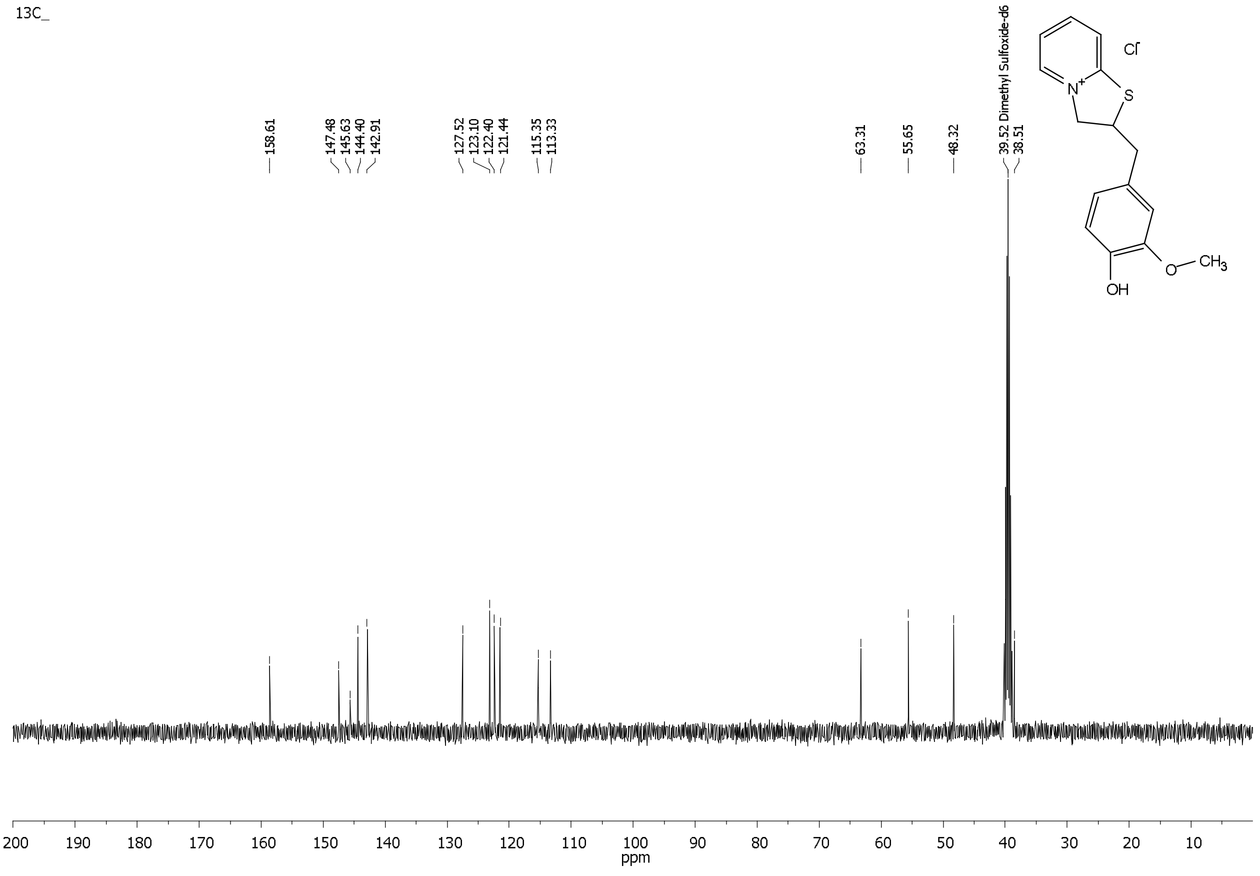

^{13}C-NMR (DMSO-d_6) spectrum of compound 2

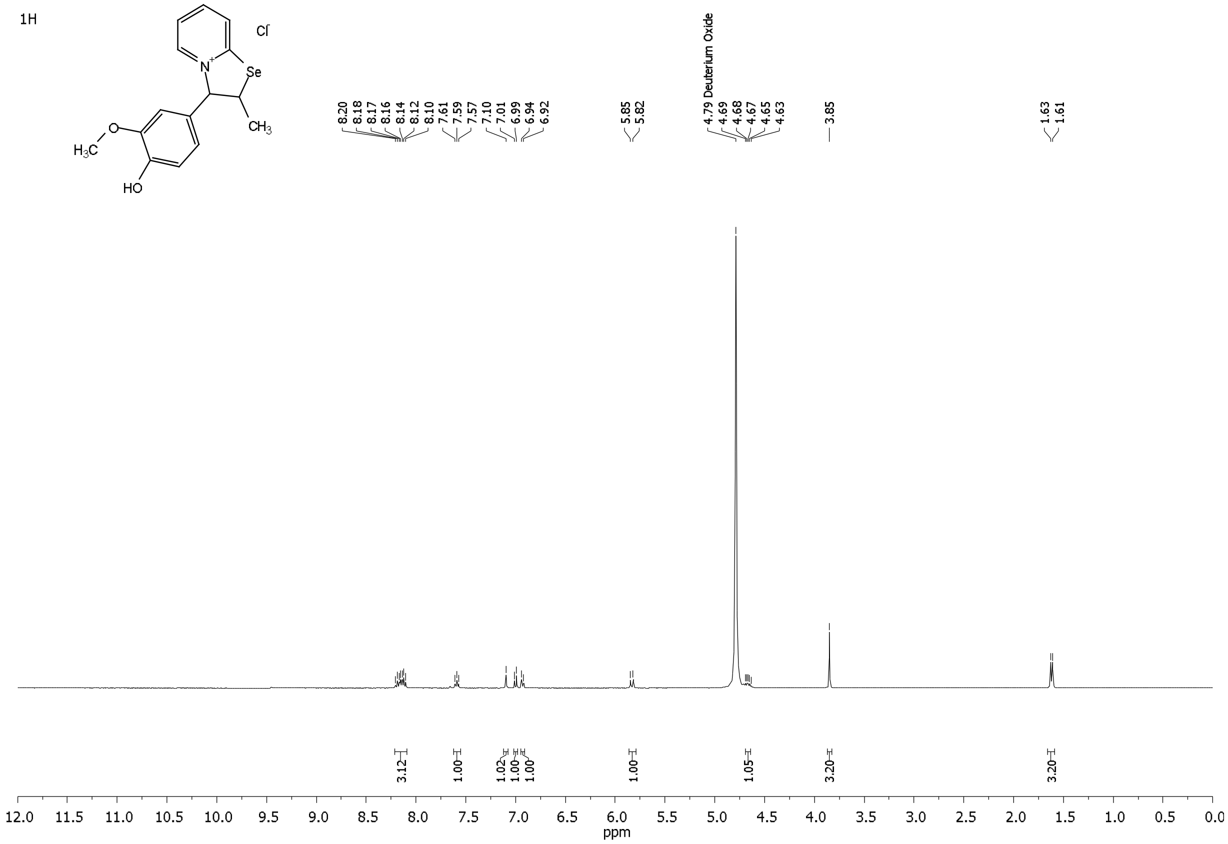

^{1}H-NMR (D_2O) spectrum of compound 3

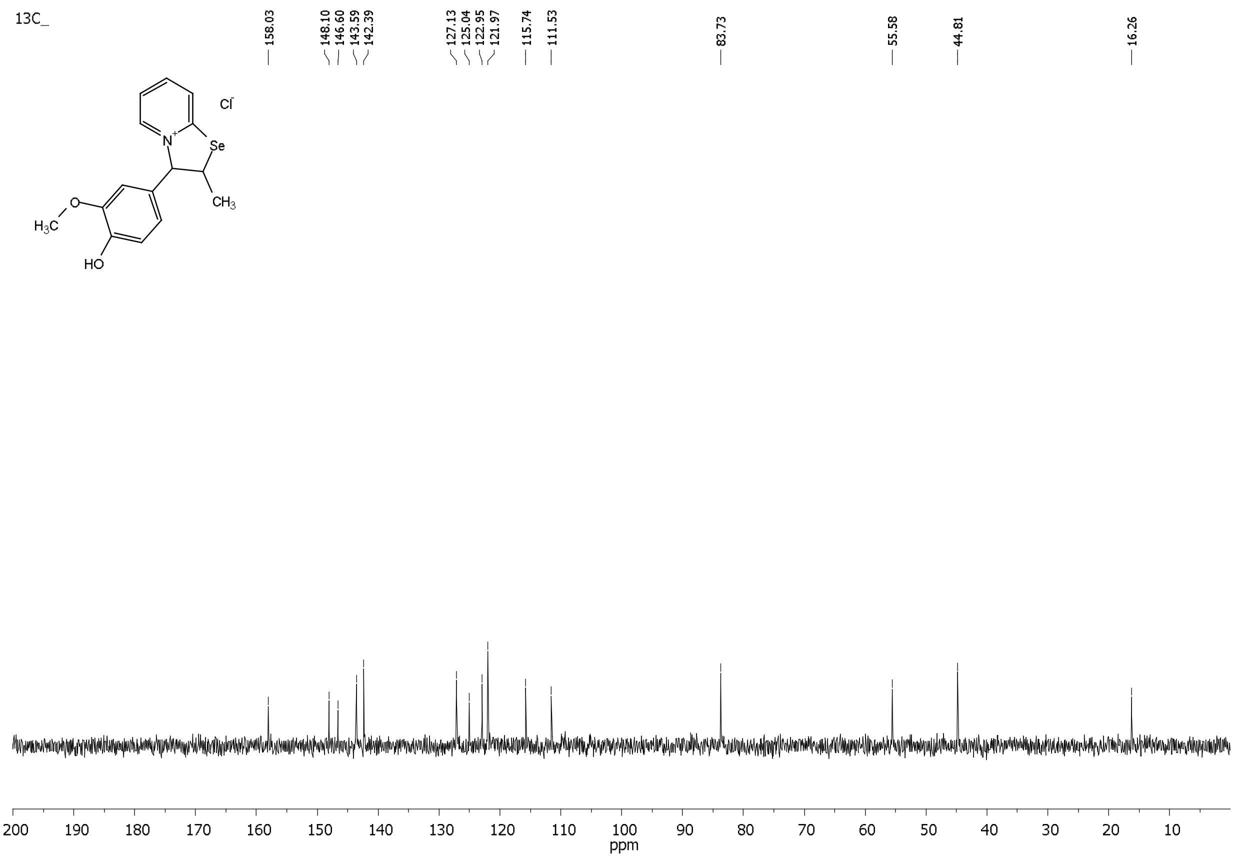

^{13}C-NMR (D_2O) spectrum of compound 3

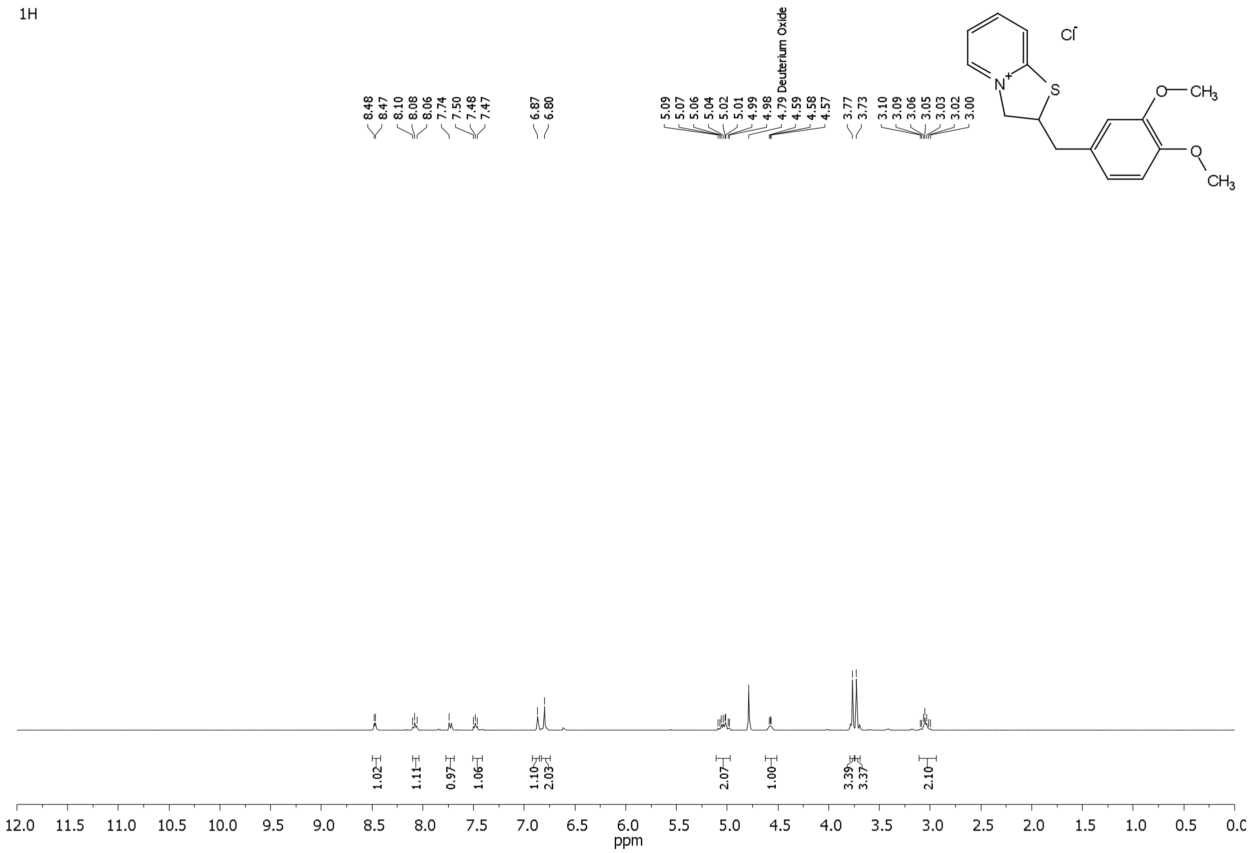

^1H-NMR (D_2O) spectrum of compound 5

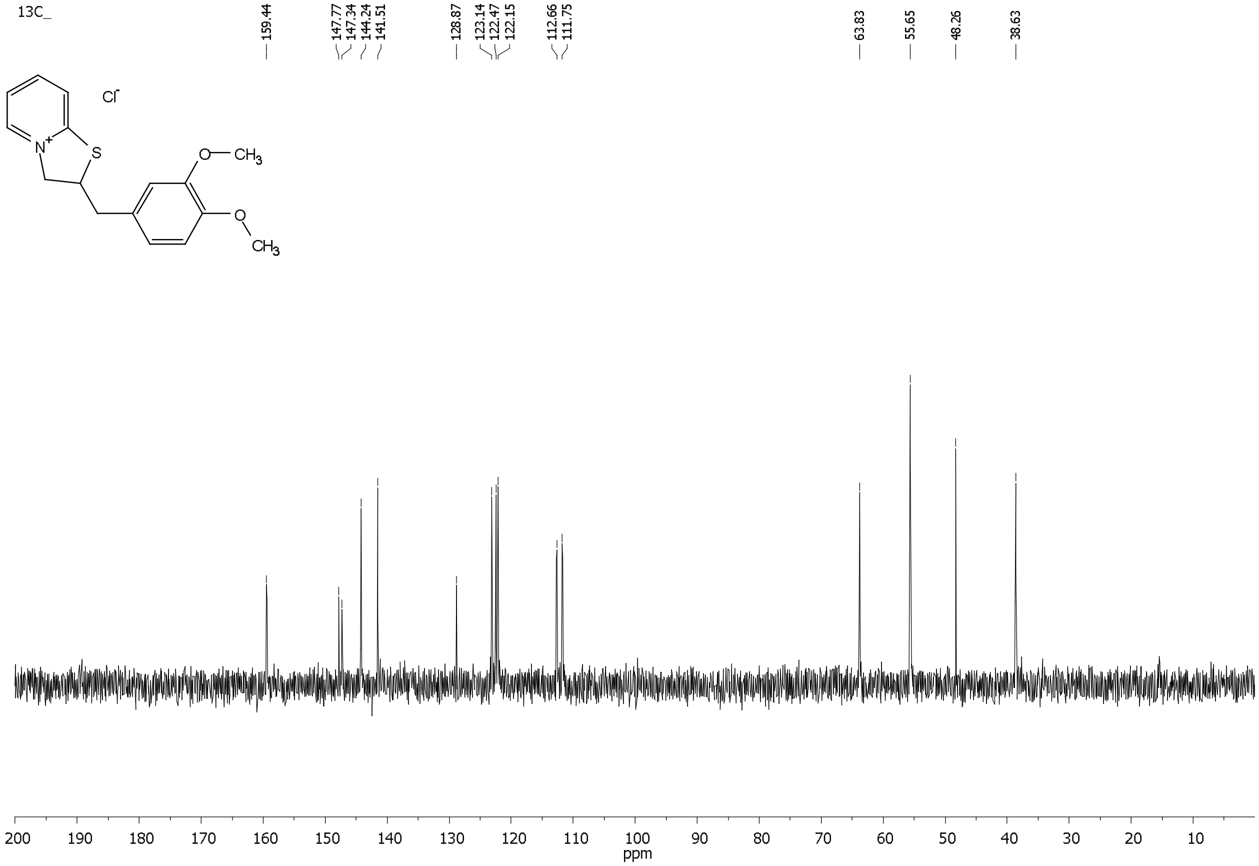

^{13}C-NMR (D_2O) spectrum of compound 5

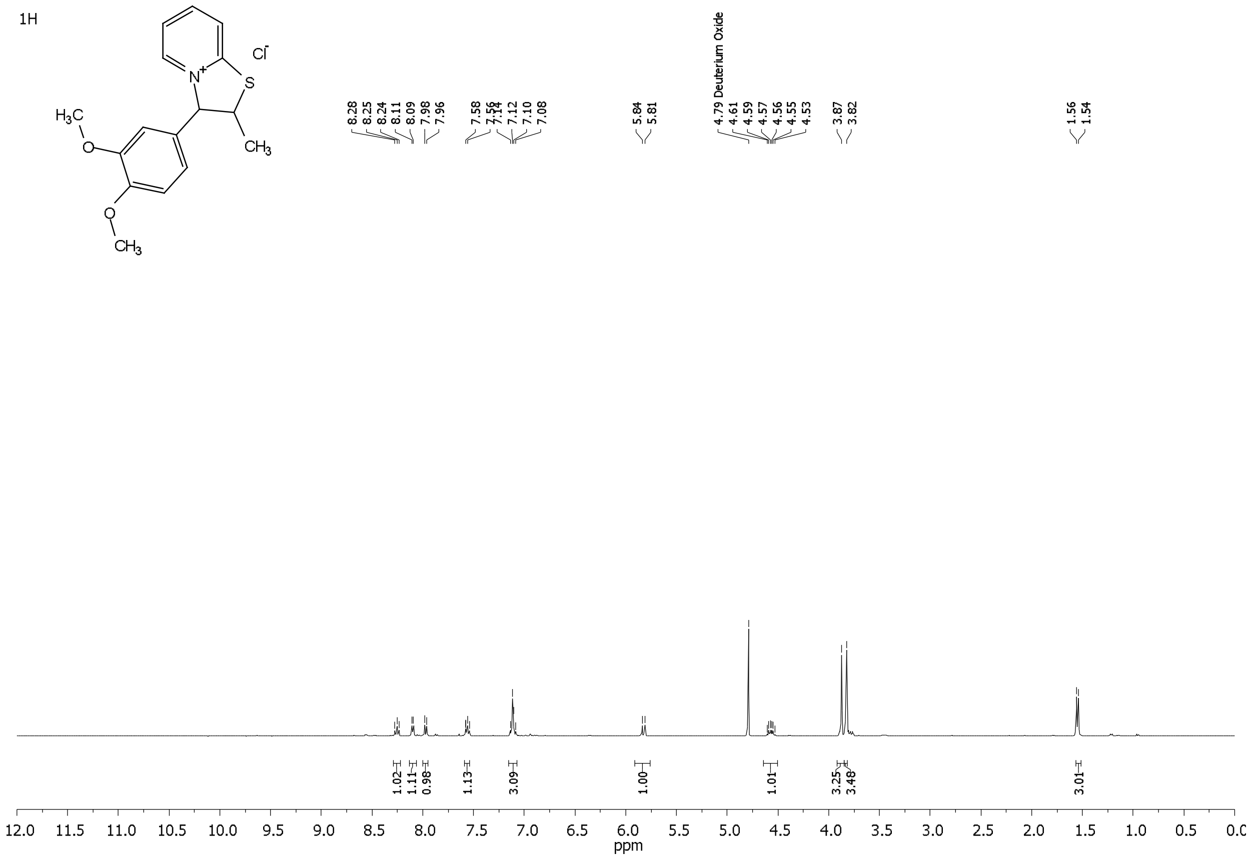

^{1}H-NMR (D_2O) spectrum of compound 6

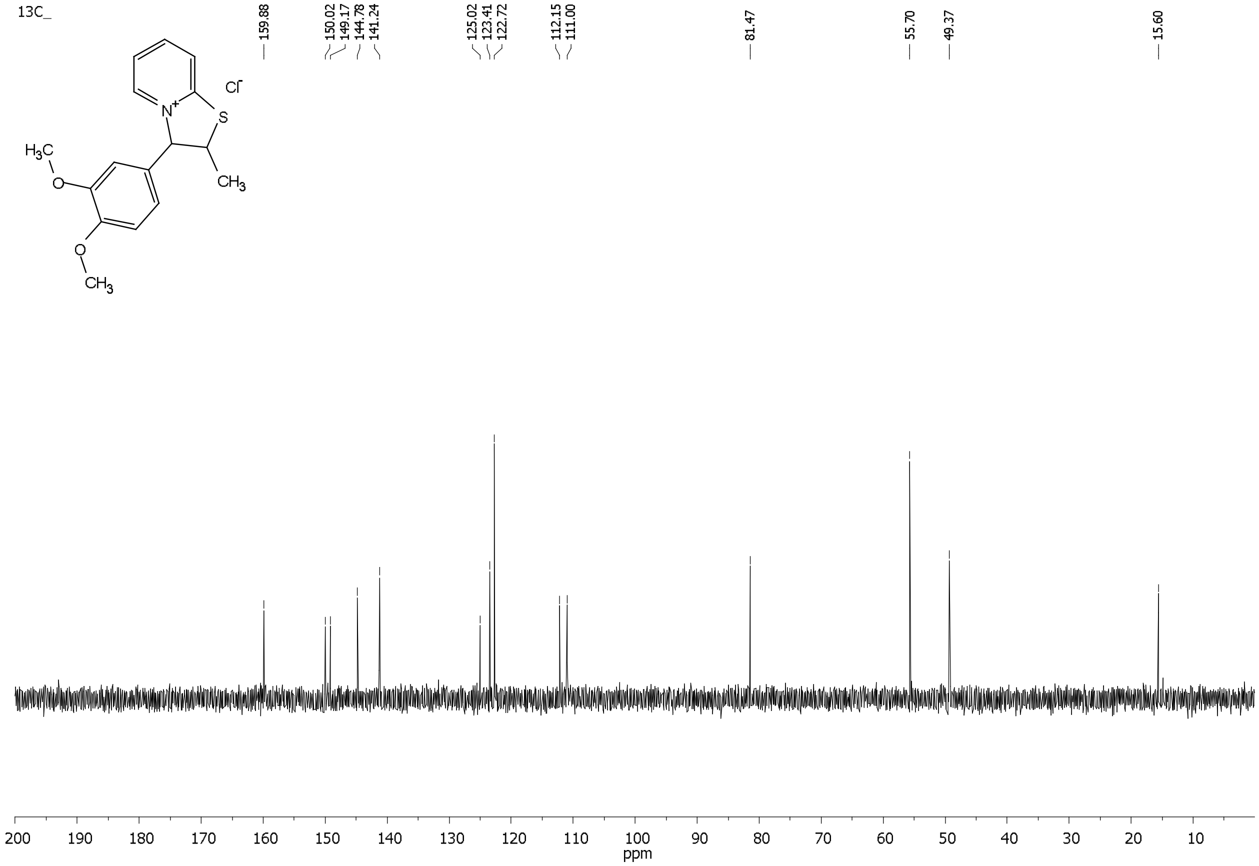

^{13}C-NMR (D_2O) spectrum of compound 6

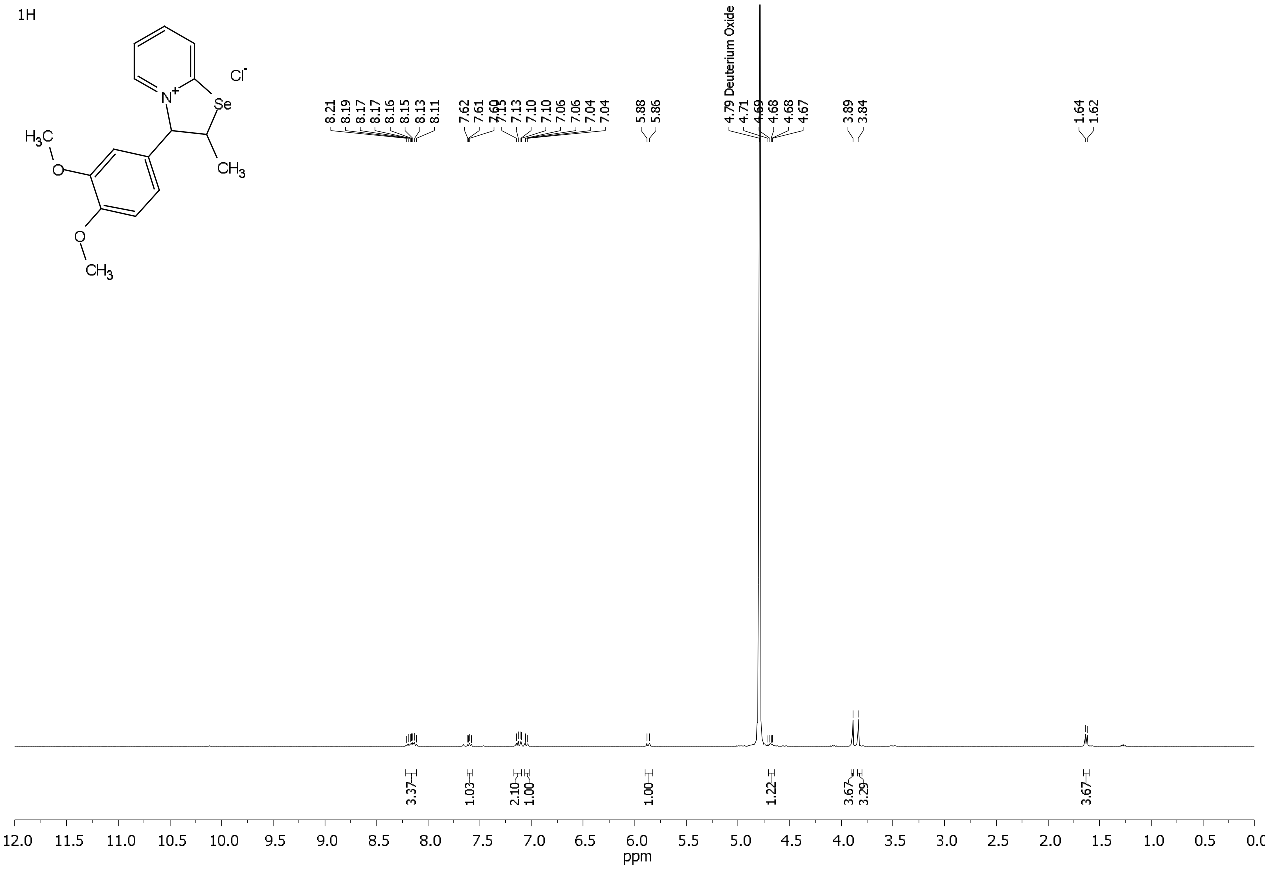

^{1}H-NMR (D_2O) spectrum of compound 8

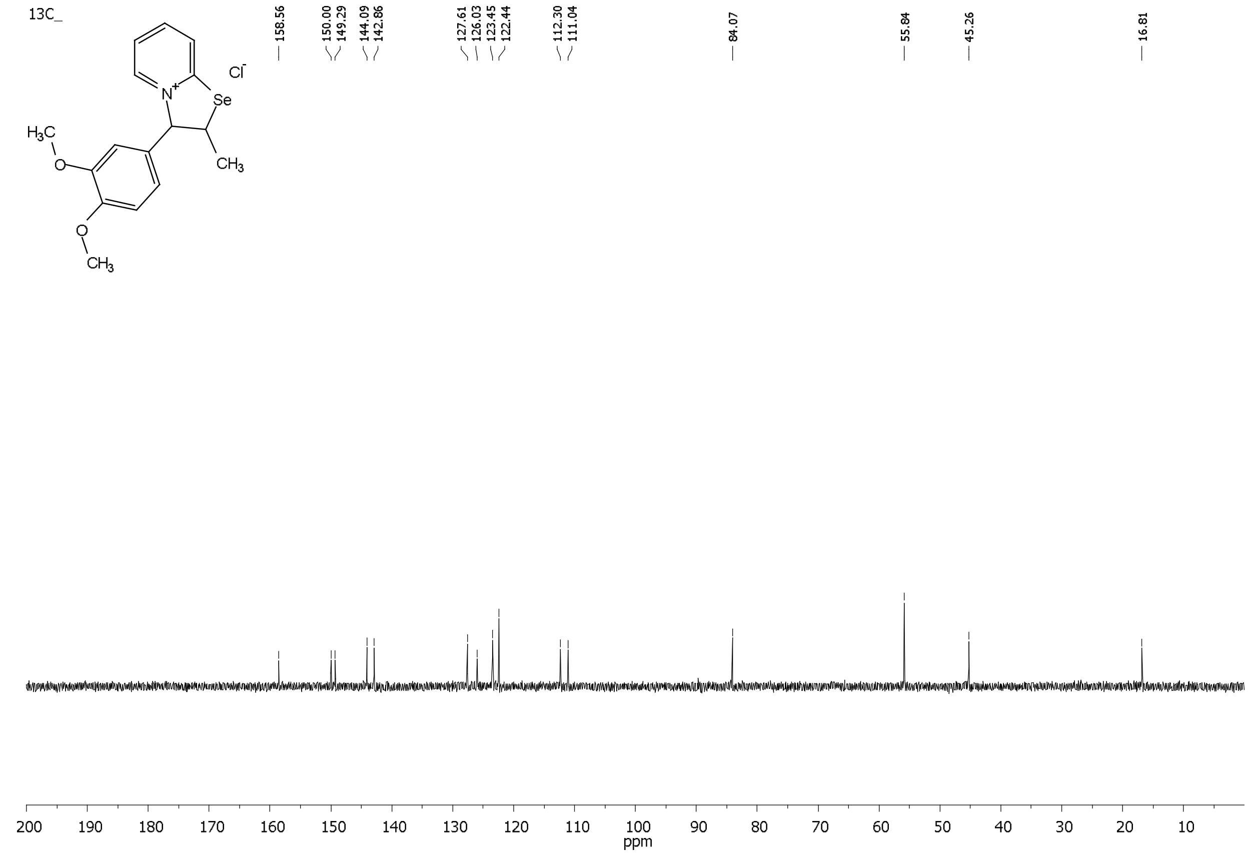

^{13}C-NMR (D_2O) spectrum of compound 8

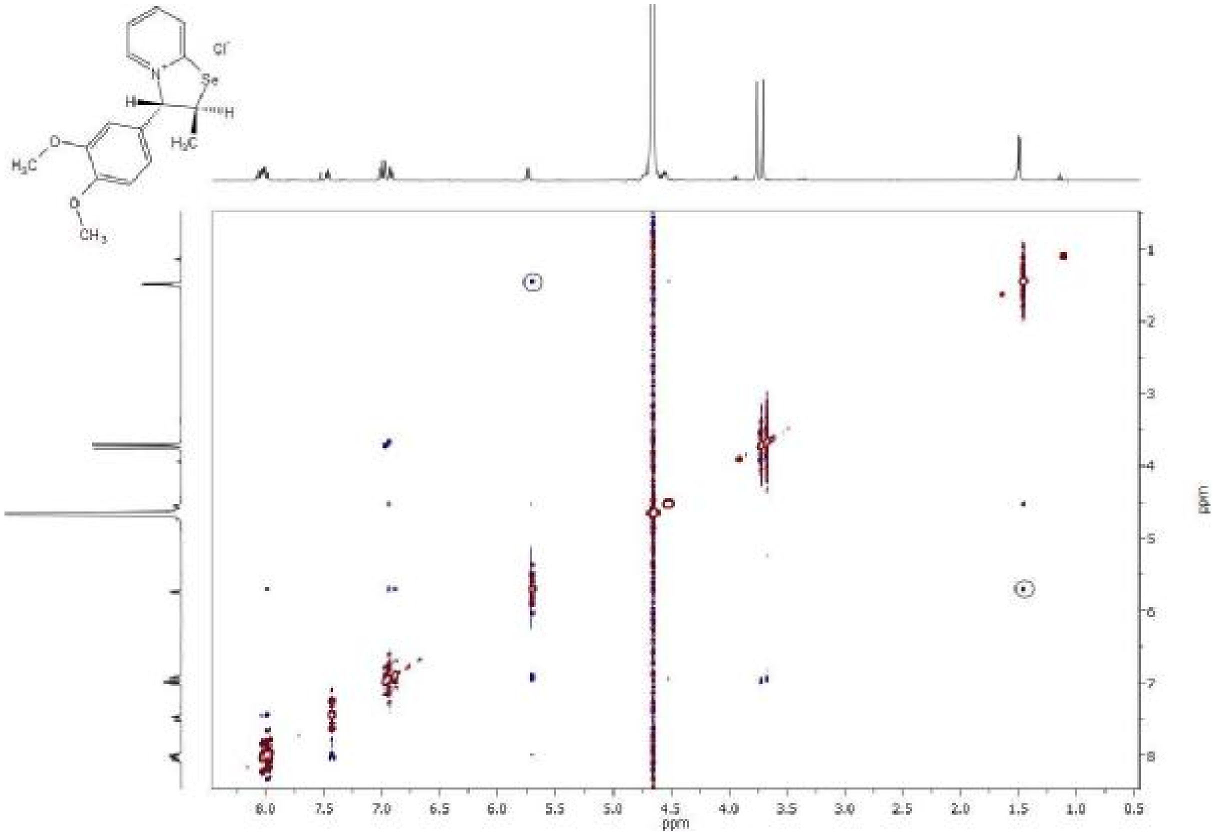

^{1}H-NMR NOESY (D_2O) spectrum of compound 8

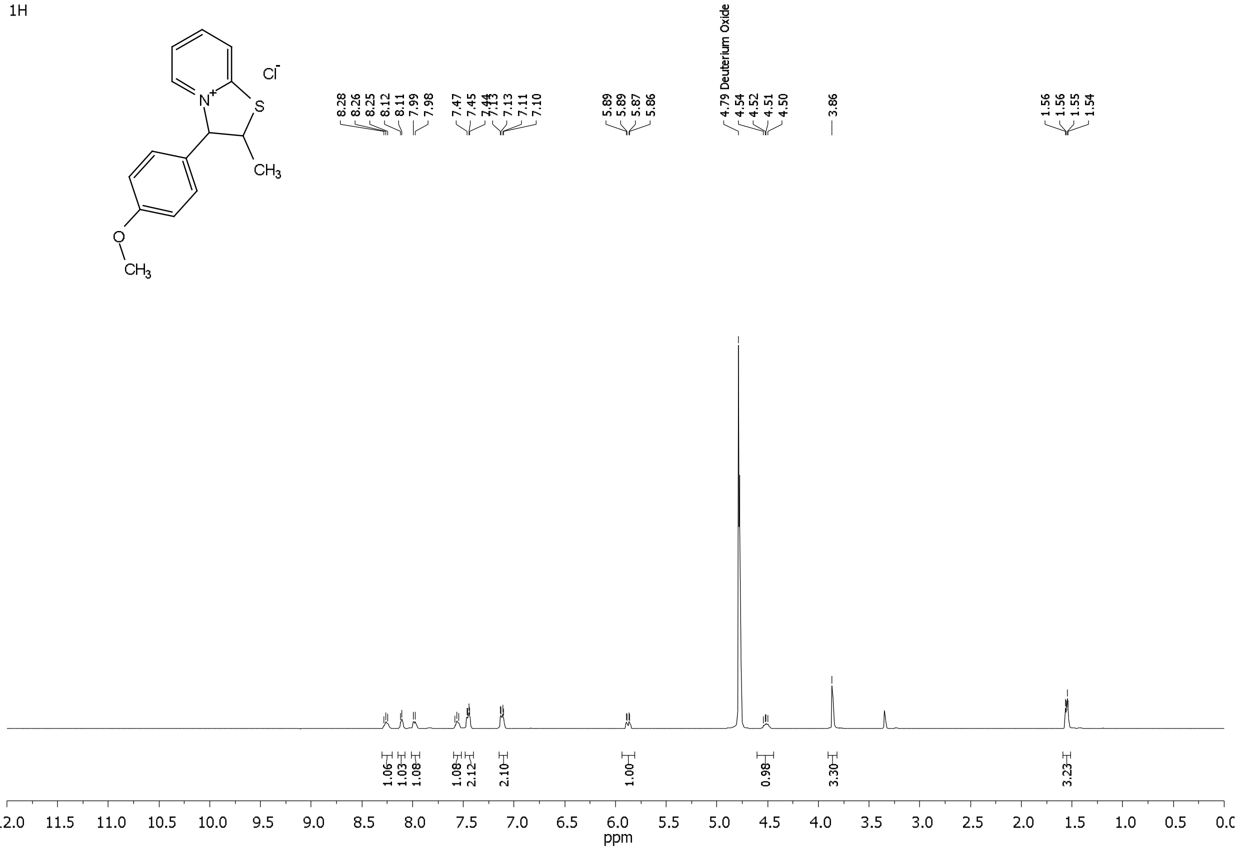

^{1}H-NMR (D_2O) spectrum of compound 11

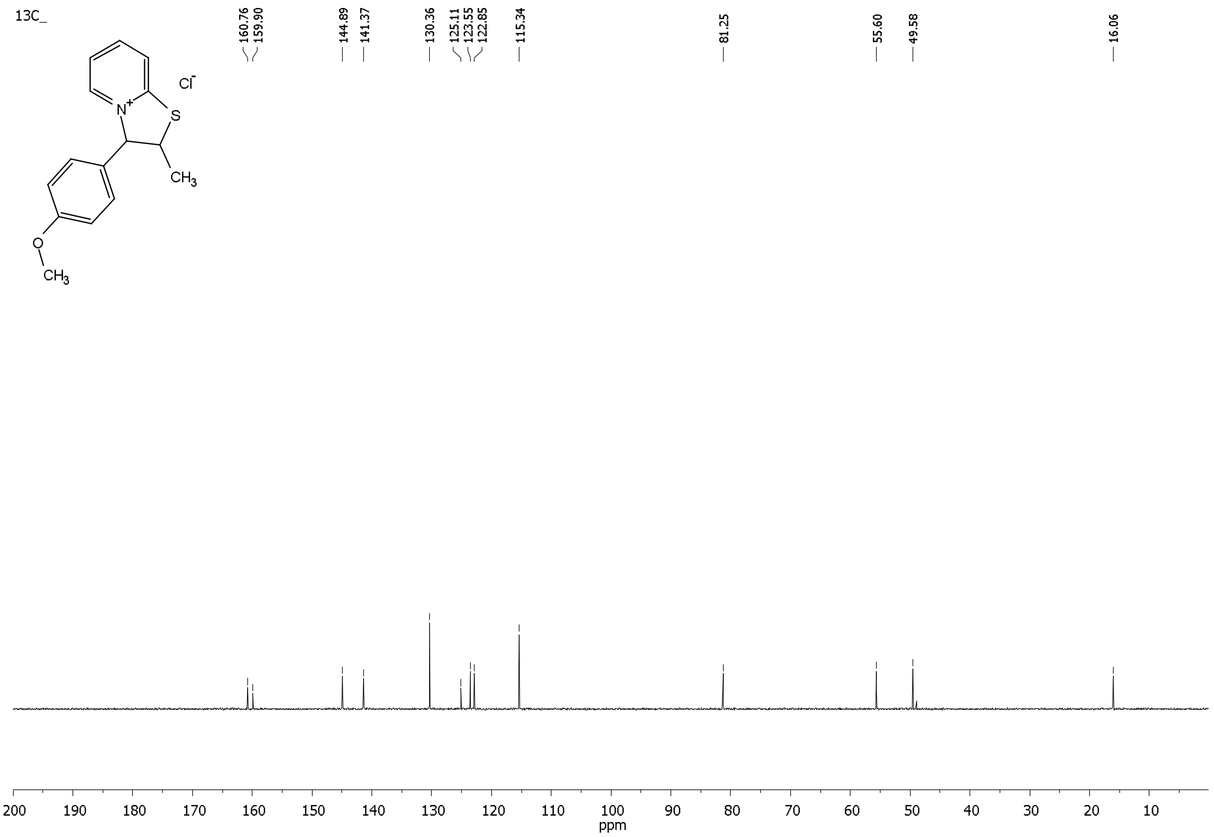

^{13}C-NMR (D_2O) spectrum of compound 11

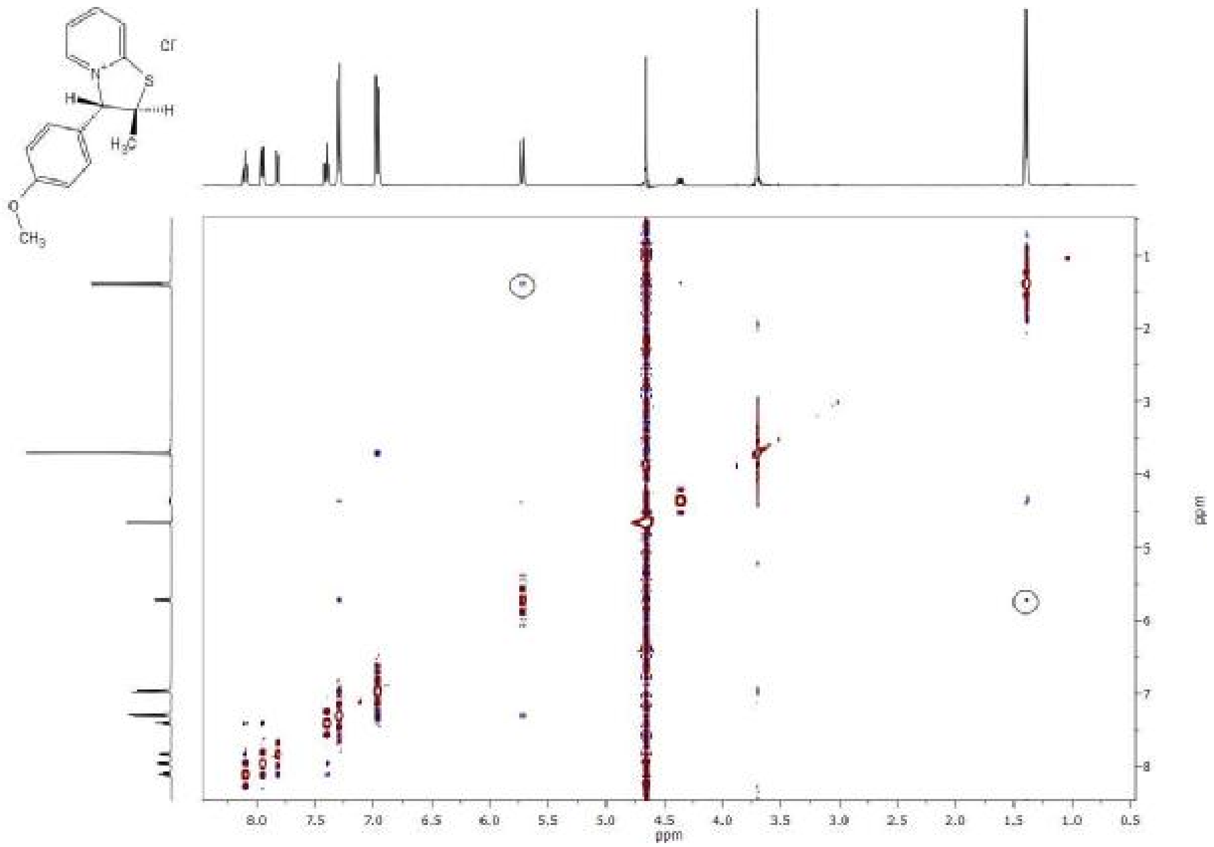

^{1}H-NMR NOESY (D_2O) spectrum of compound 11

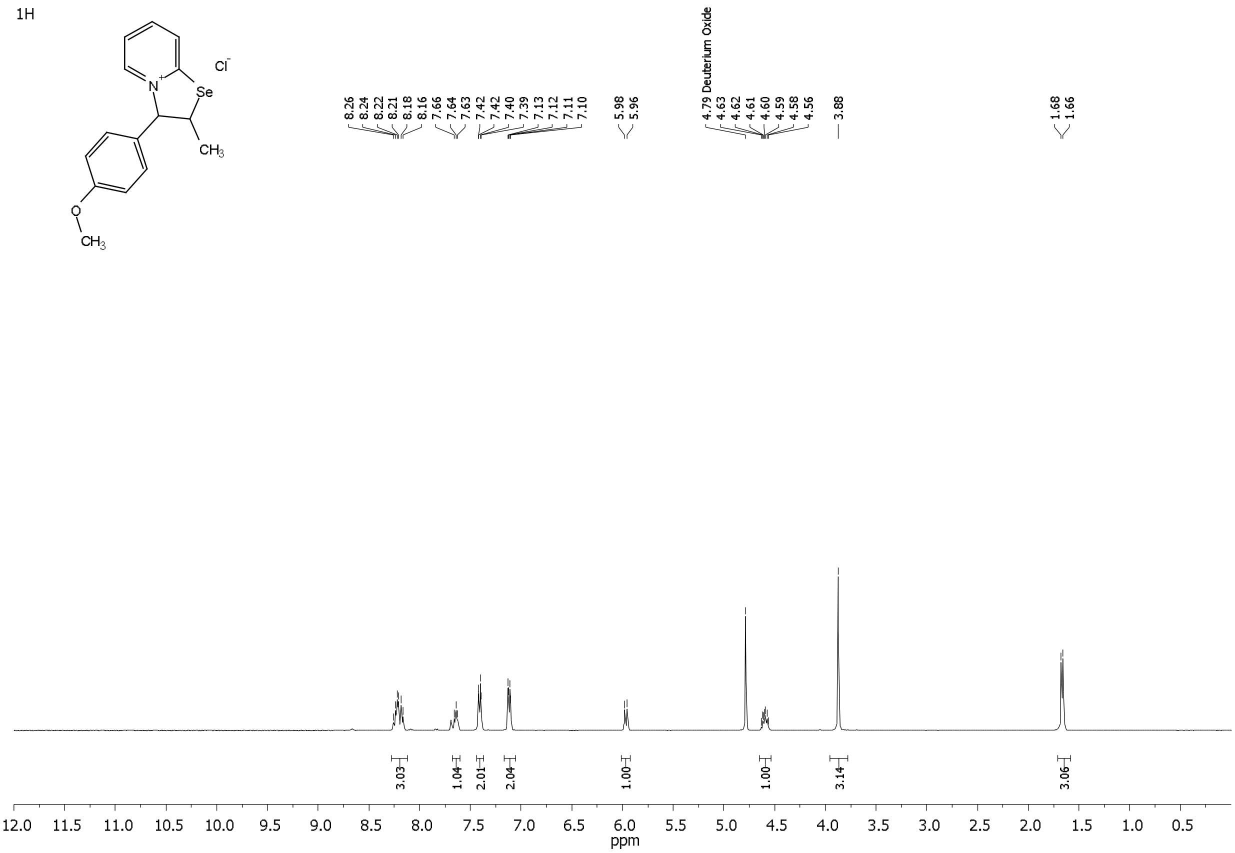

^{1}H-NMR (D_2O) spectrum of compound 12

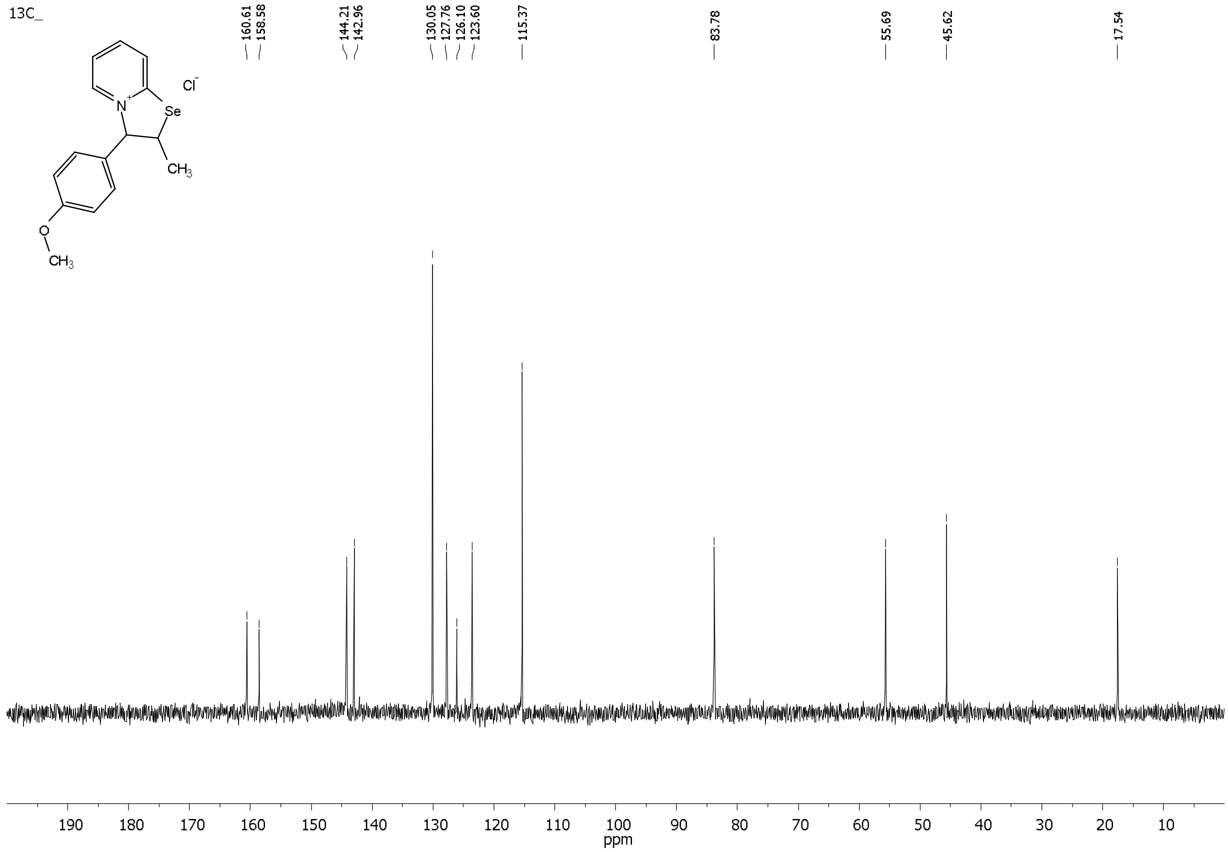

^{13}C-NMR (D_2O) spectrum of compound 12

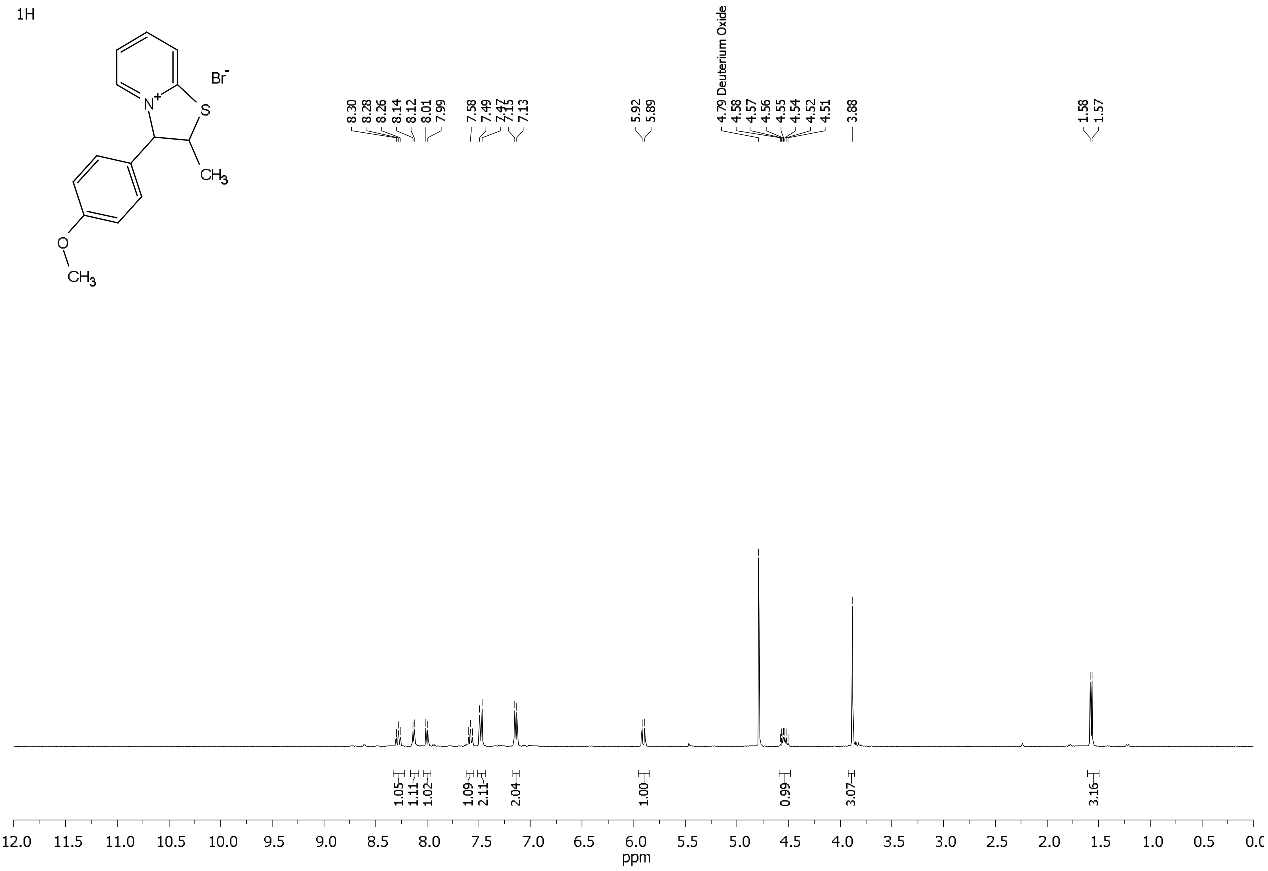

^{1}H-NMR (D_2O) spectrum of compound 13

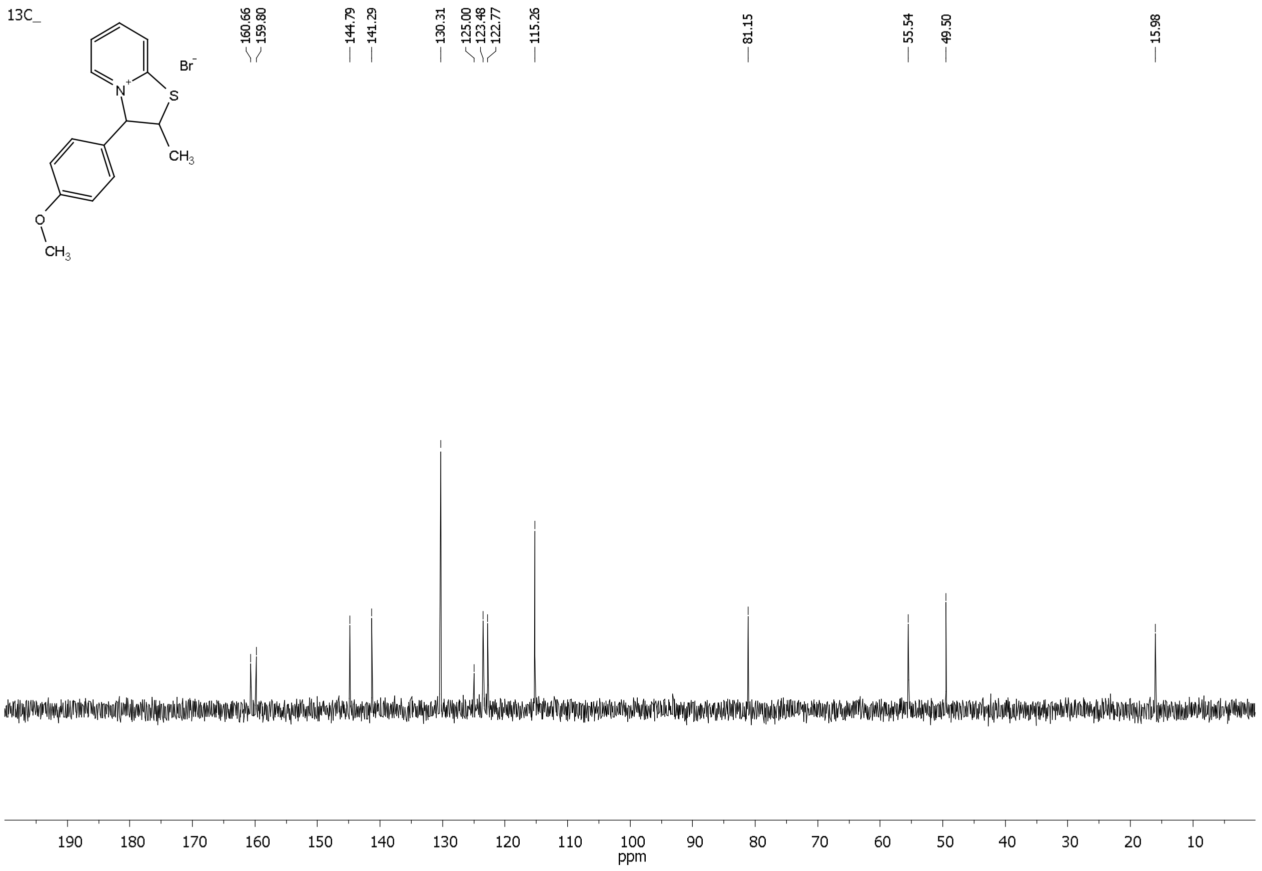

^{13}C-NMR (D_2O) spectrum of compound 13

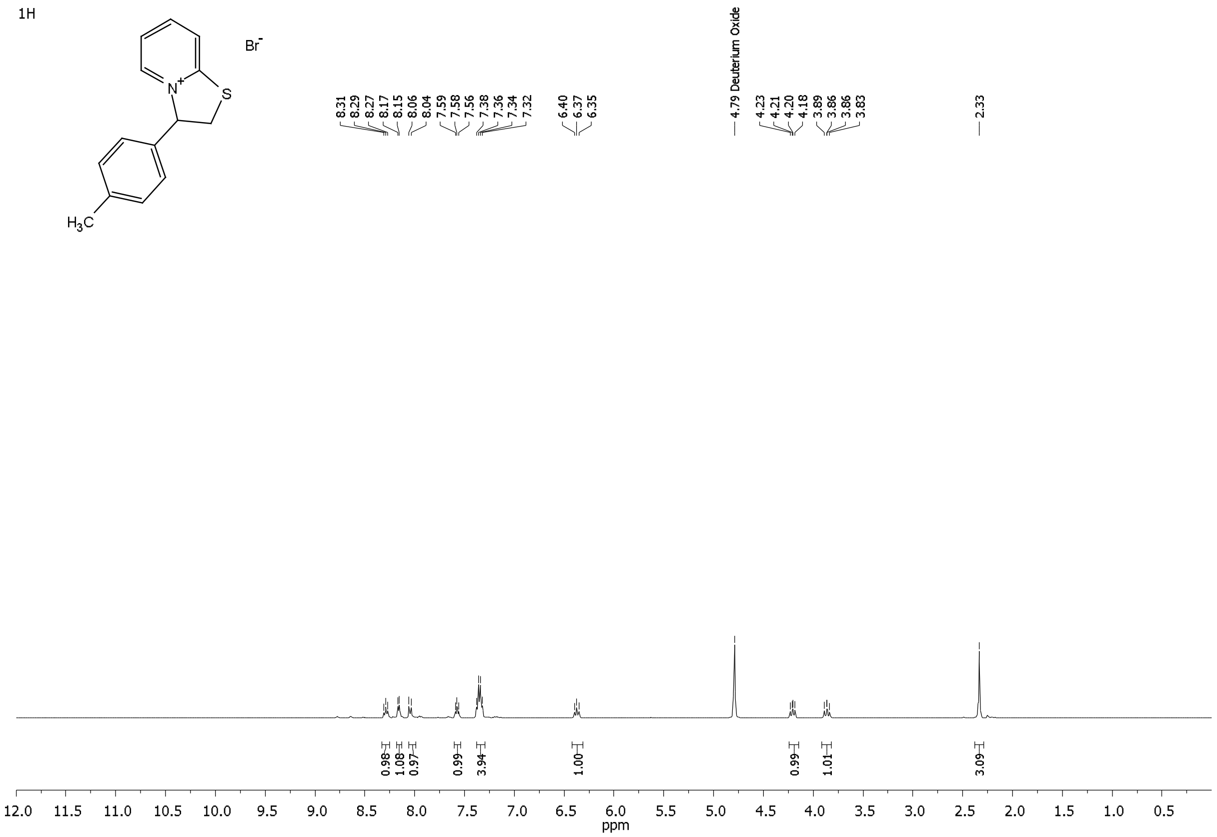

^{1}H-NMR (D_2O) spectrum of compound 14

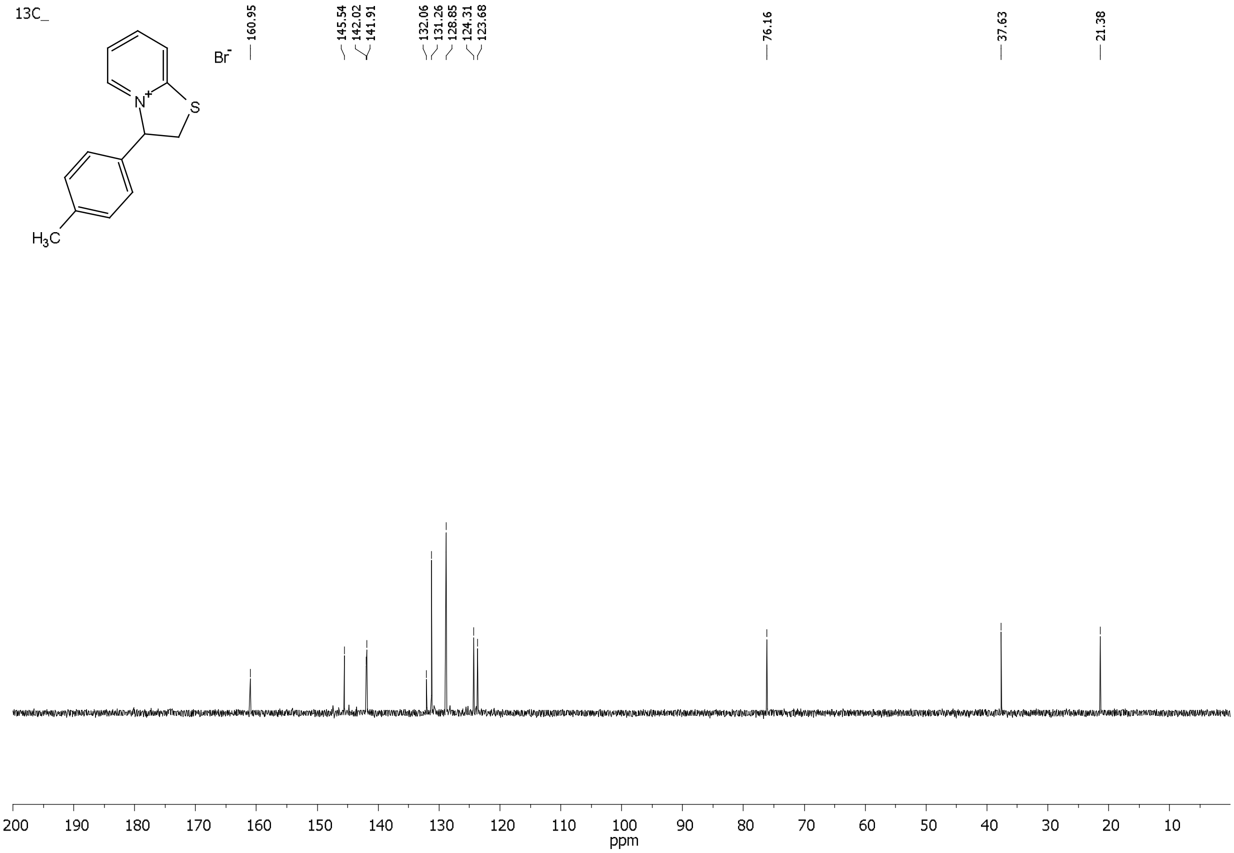

^{13}C-NMR (D_2O) spectrum of compound 14

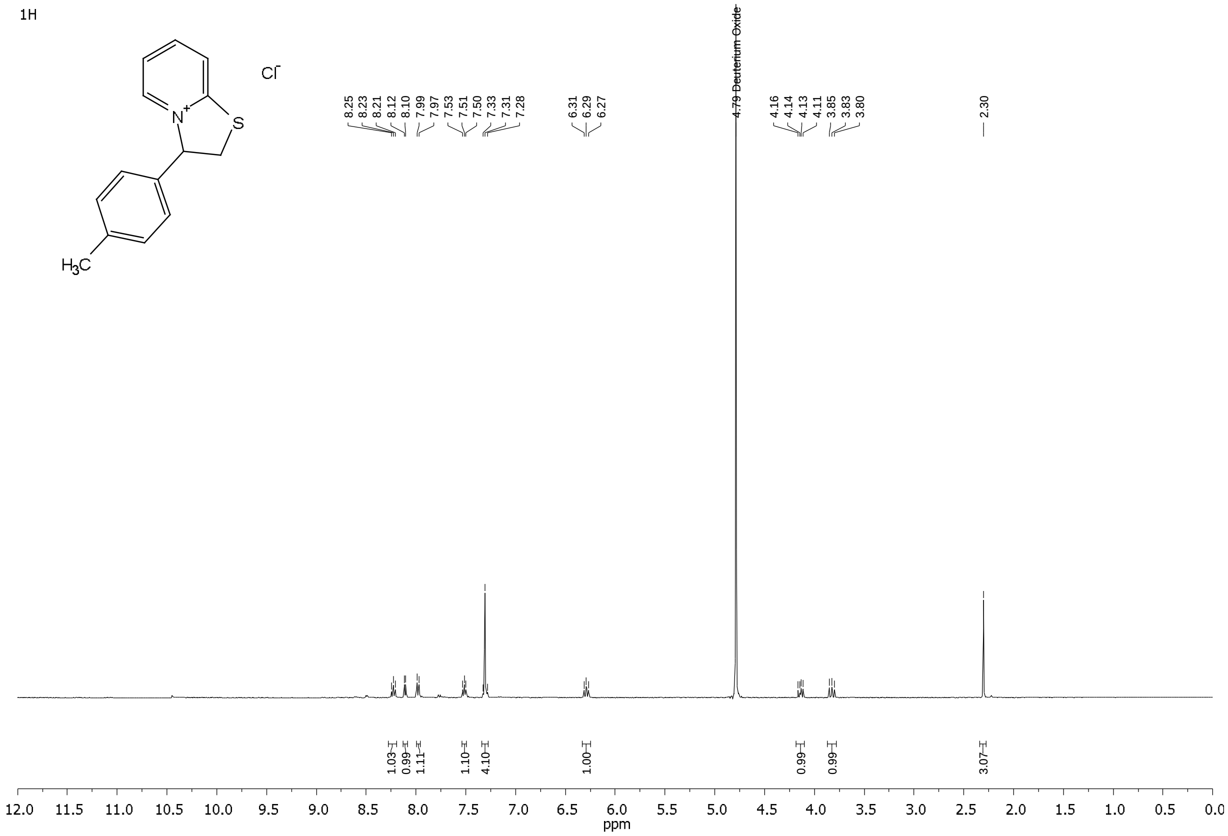

^{1}H-NMR (D_2O) spectrum of compound 15

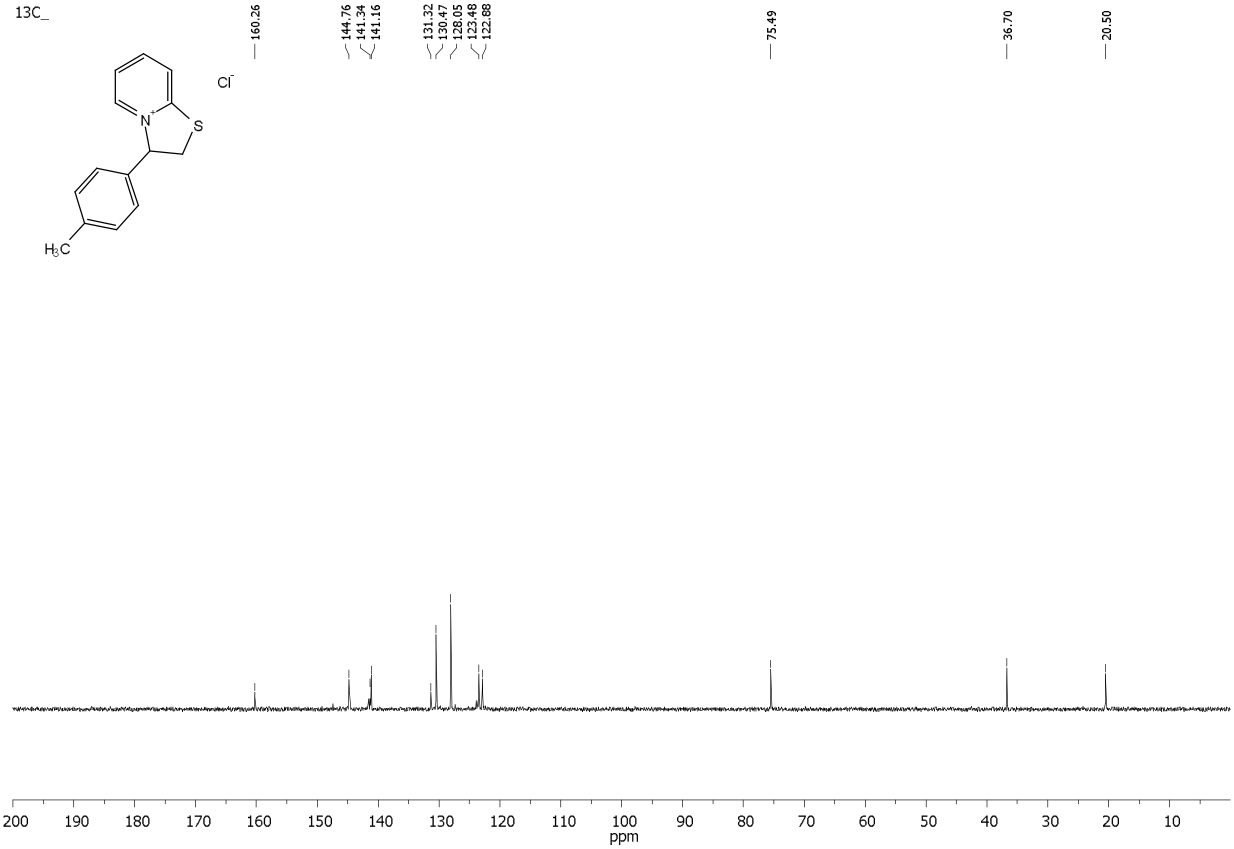

^{13}C-NMR (D_2O) spectrum of compound 15

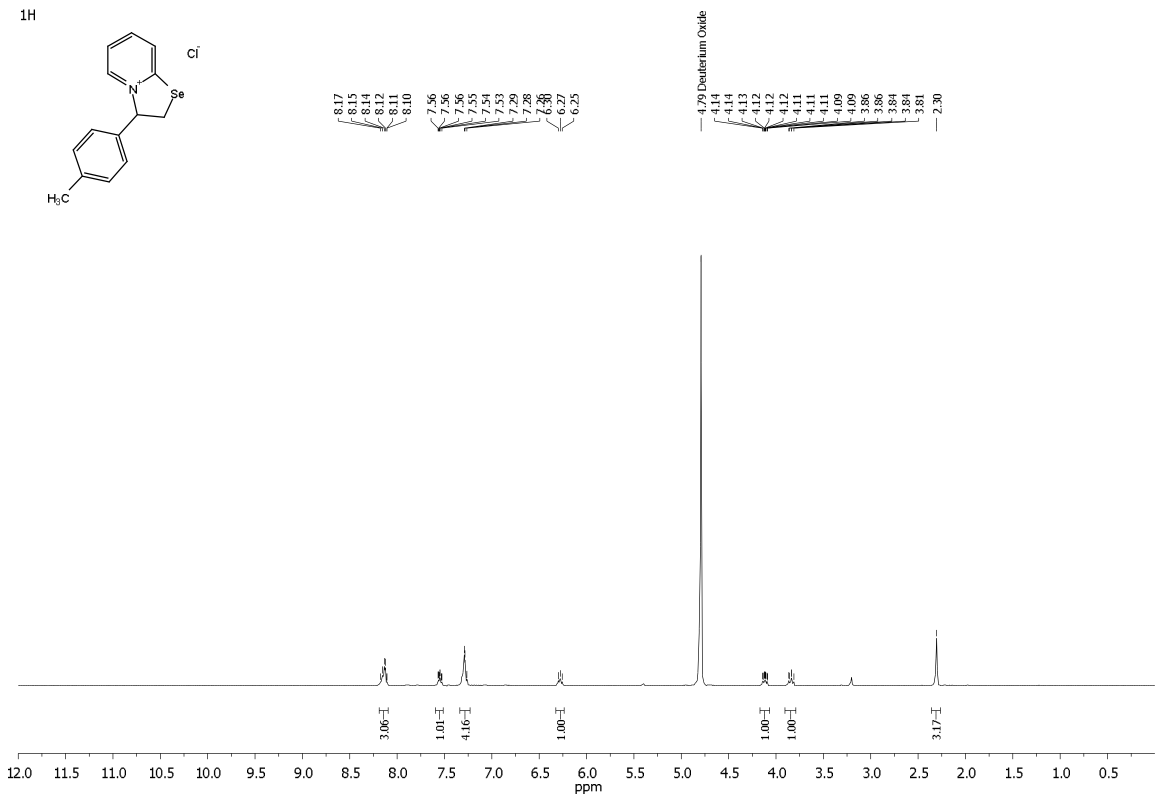

^{1}H-NMR (D_2O) spectrum of compound 17

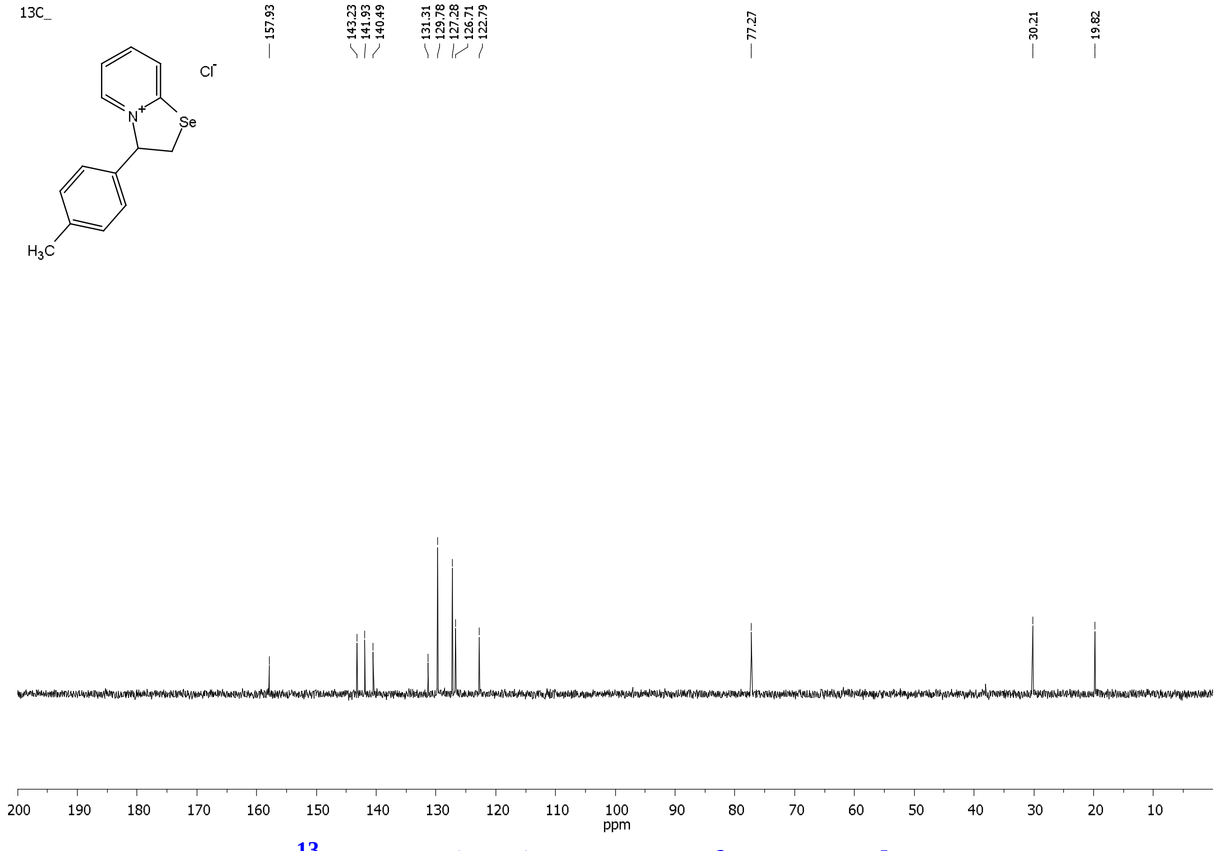

^{13}C-NMR (D_2O) spectrum of compound 17

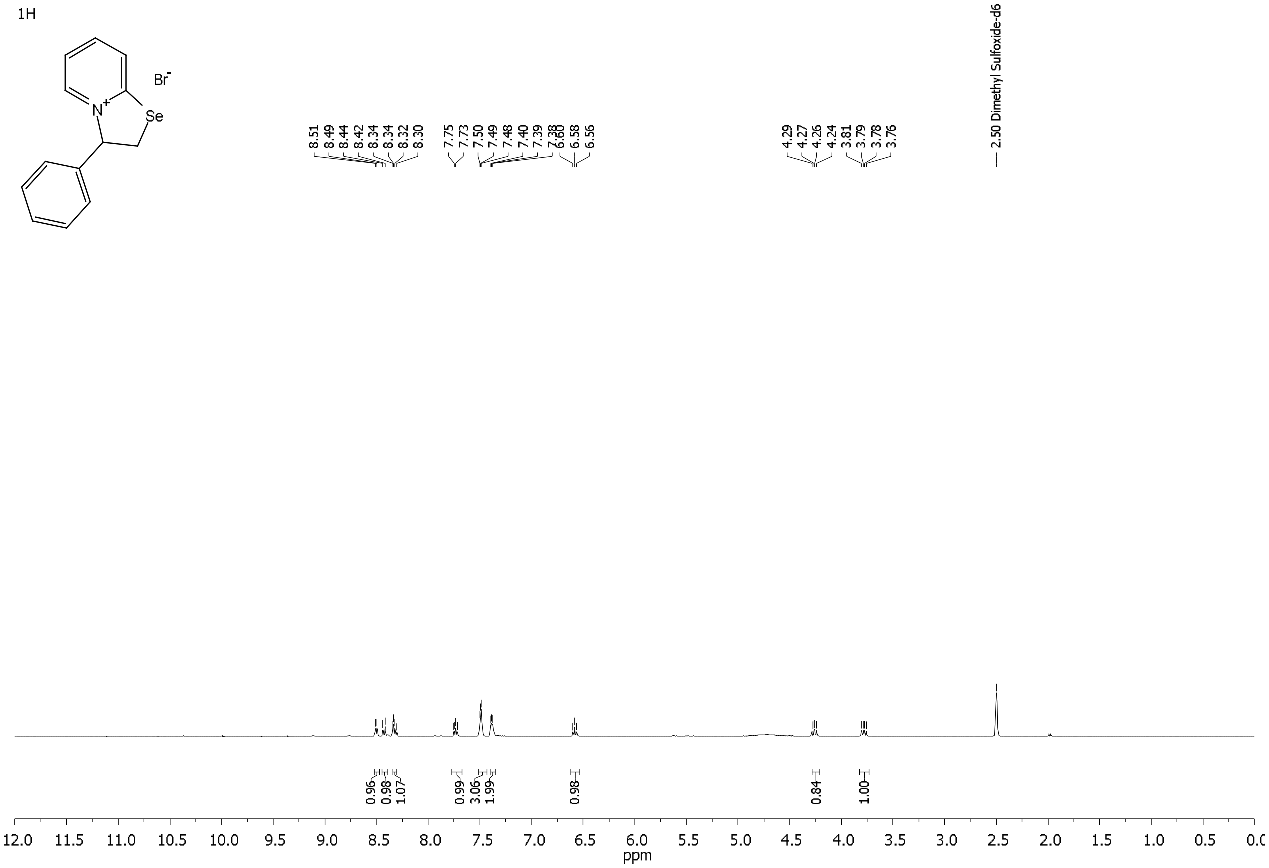

^{1}H-NMR (DMSO-d_6) spectrum of compound 18

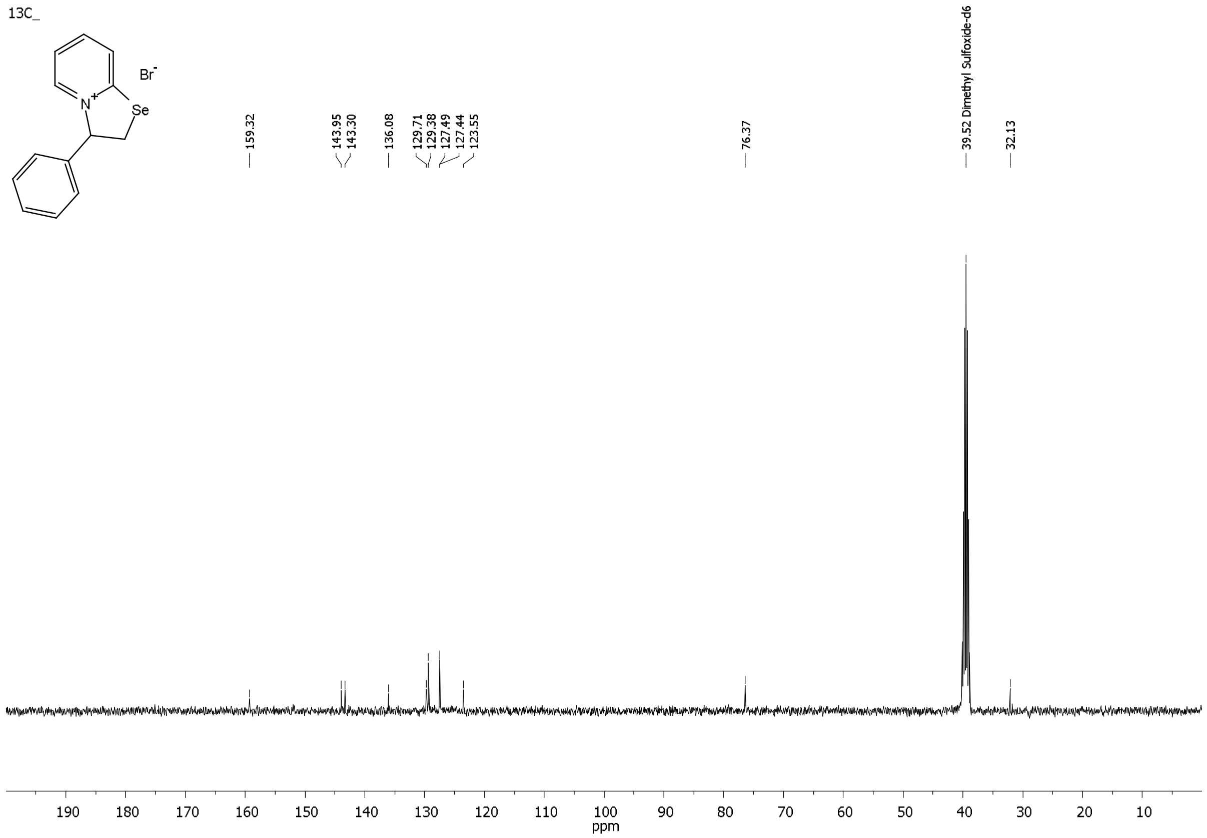

^{13}C-NMR (DMSO-d_6) spectrum of compound 18

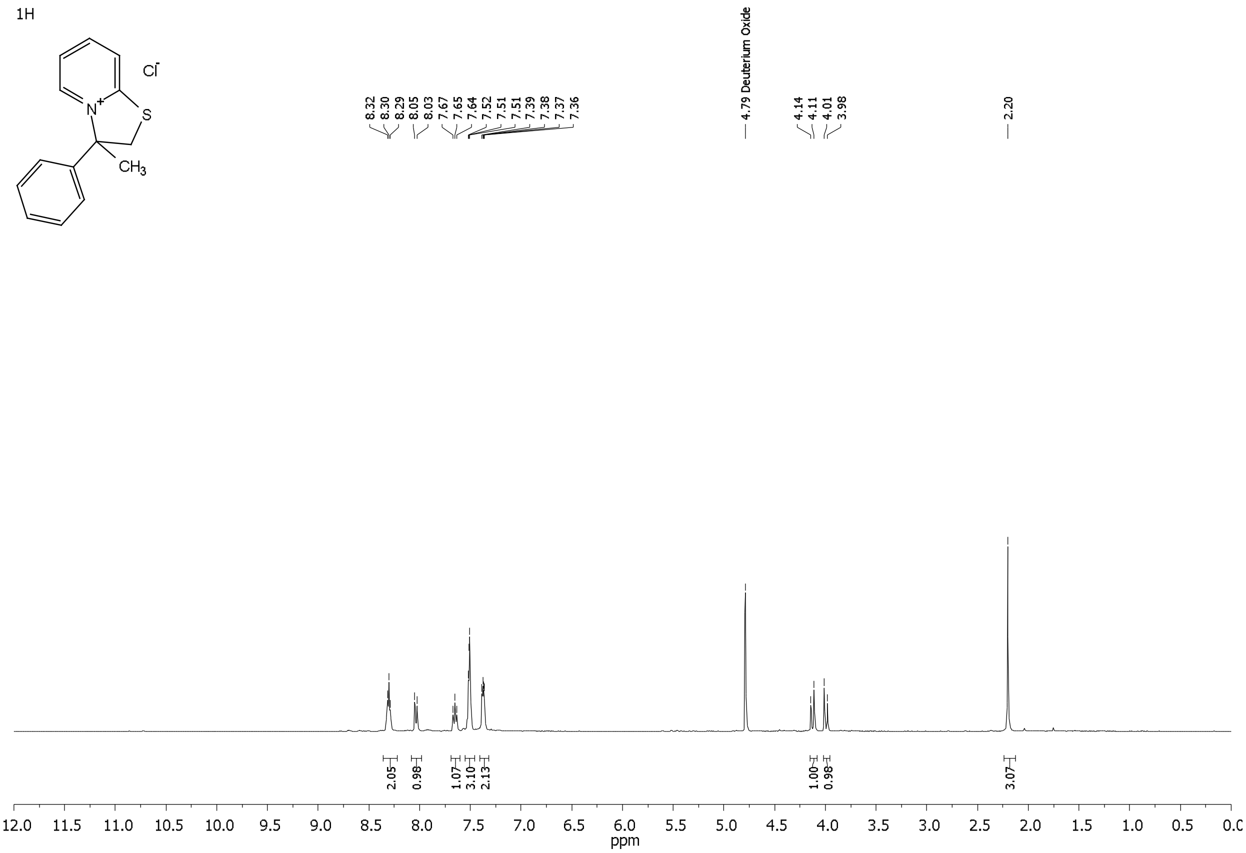

^{1}H-NMR (D_2O) spectrum of compound 22

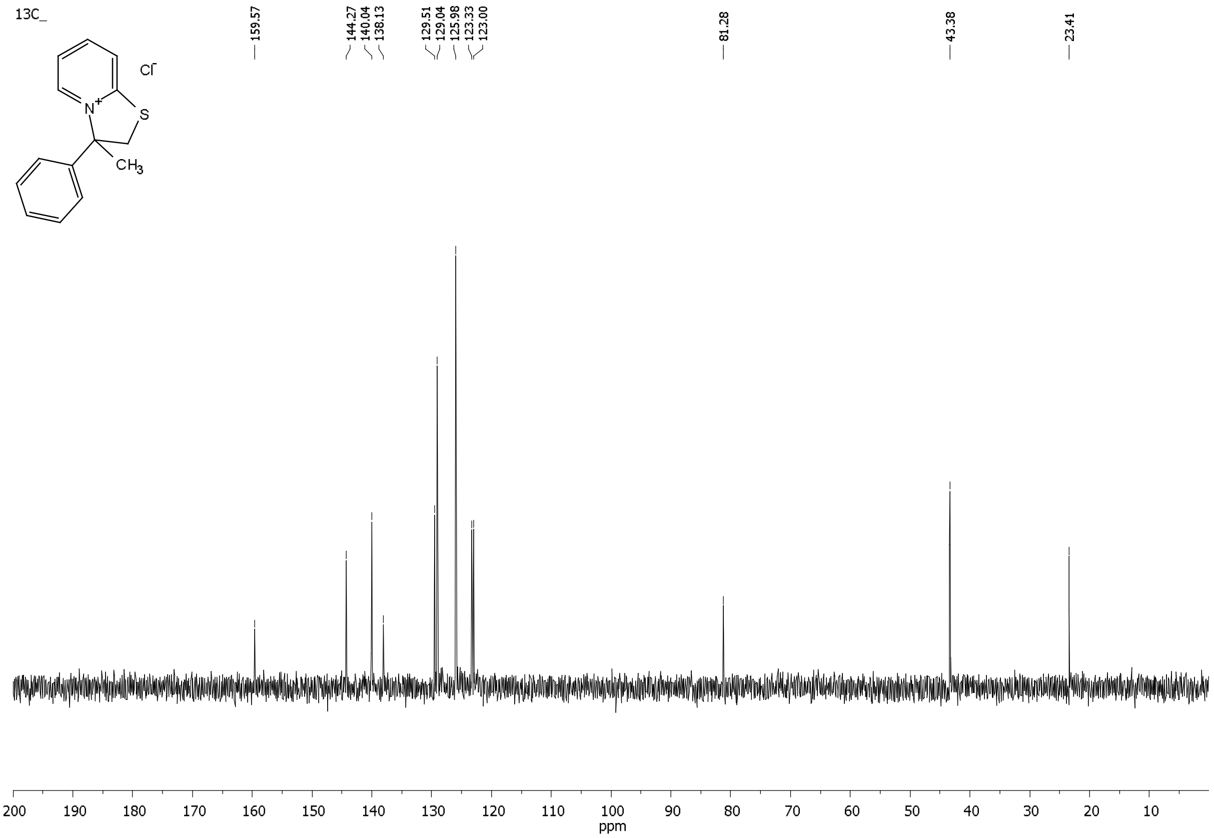

^{13}C-NMR (D_2O) spectrum of compound 22

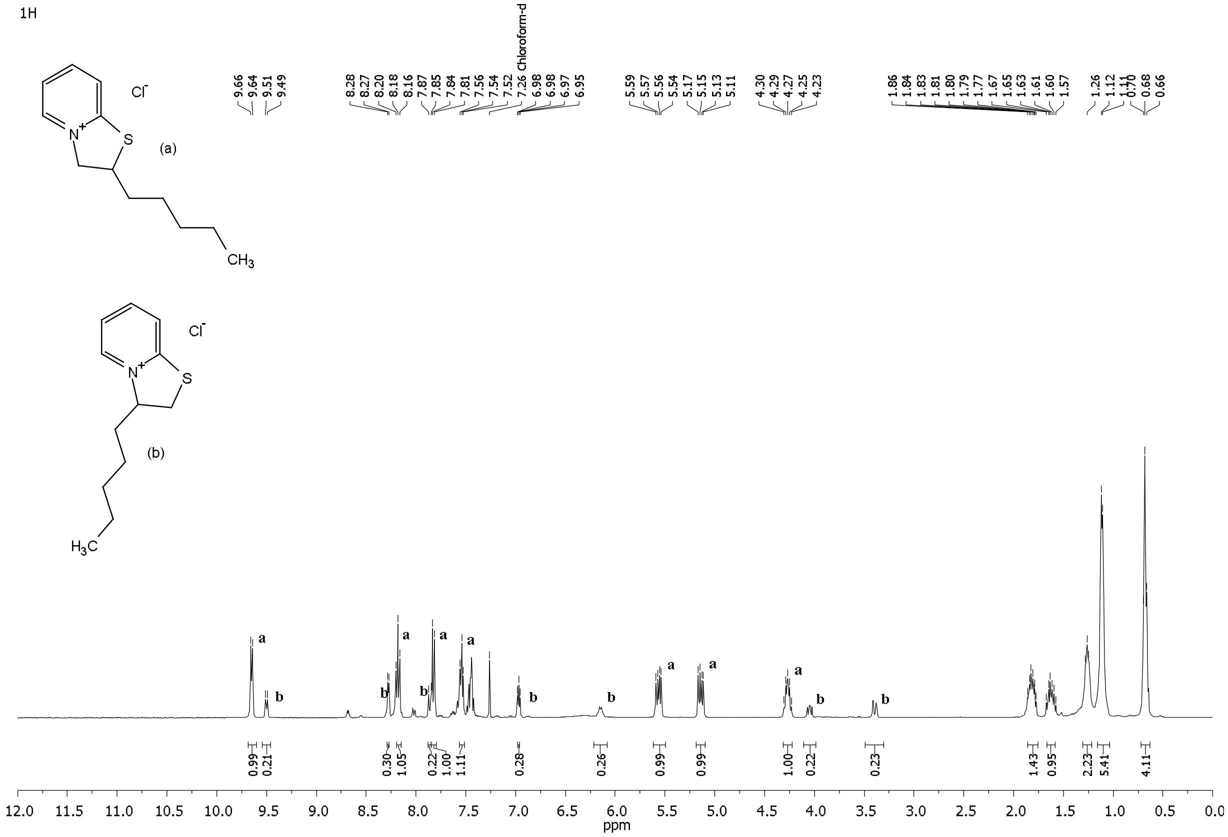

^{1}H-NMR ($CDCl_3$) spectrum of the mixture of compounds 24 (a) and 25 (b)

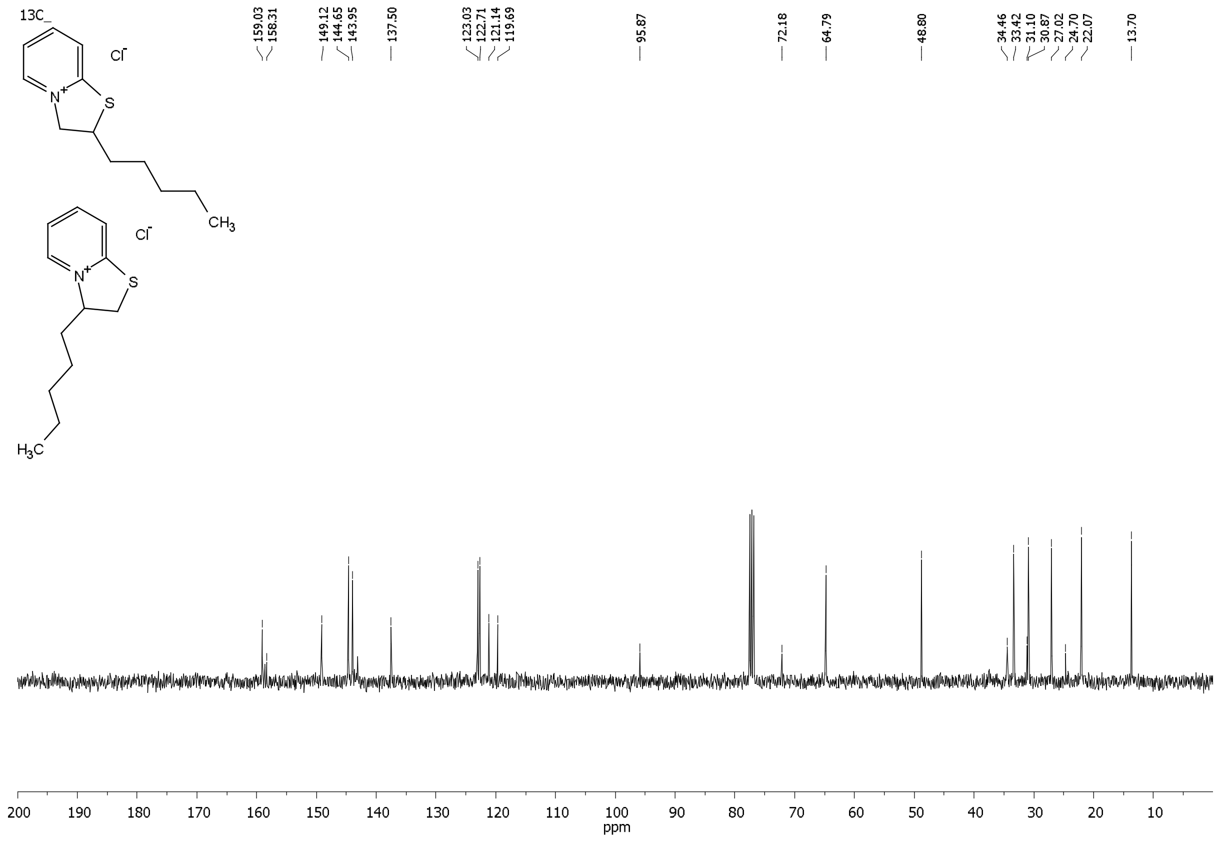

^{13}C-NMR ($CDCl_3$) spectrum of the mixture of compounds 24 and 25
